# The effects of inclusion on academic achievement, socioemotional development and wellbeing of children with special educational needs

**DOI:** 10.1002/cl2.1291

**Published:** 2022-12-07

**Authors:** Nina T. Dalgaard, Anja Bondebjerg, Bjørn C. A. Viinholt, Trine Filges

**Affiliations:** ^1^ VIVE—The Danish Centre for Social Science Research Copenhagen Denmark

## Abstract

**Background:**

Considering the rapid global movement towards inclusion for students with special educational needs (SEN), there is a surprising lack of pedagogical or didactic theories regarding the ways in which inclusive education may affect students with SEN. Group composition within the educational setting may play a role in determining the academic achievement, socio‐emotional development, and wellbeing of students with SEN. Proponents of inclusion propose that segregated educational placement causes stigmatisation and social isolation which may have detrimental effects on the self‐concept and self‐confidence of students with SEN. On the other hand, opponents of inclusion for all special needs students suggest that placement in general education classrooms may have adverse effects especially if the time and resources allocated for individualisation are not aligned with student needs. Since the 1980s, a number of reviews on the effects of inclusion have been published. Results are inconsistent, and several reviews point to a number of methodological challenges and weaknesses of the study designs within primary studies. In sum, the impact of inclusion on students with SEN may be hypothesised to be both positive and negative, and the current knowledge base is inconsistent.

**Objectives:**

The objective was first:

- To uncover and synthesise data from contemporary studies to assess the effects of inclusion on measures of academic achievement, socio‐emotional development, and wellbeing of children with special needs when compared to children with special needs who receive special education in a segregated setting.
- A secondary objective was to explore how potential moderators (gender, age, type and severity of special need, part or full time inclusive education, and co‐teaching) relate to outcomes.

**Search Methods:**

Relevant studies were identified through electronic searches in Academic Search Premier (EBSCO), APA PsycINFO (EBSCO), EconLit (EBSCO), ERIC (EBSCO), International Bibliography of the Social Sciences (ProQuest), Sociological Abstracts (ProQuest), Science Citation Index Expanded (Web Of Science), Social Sciences Citation Index (Web Of Science), and SocINDEX (EBSCO). The database searches were completed on 24 April 2021 and other resources: grey literature repositories, hand search in targeted journals and Internet search engines were searched in August/September 2021. The search was limited to studies reported after 2000.

**Selection Criteria:**

The review included studies of children with special needs in grades K to 12 in the OECD countries. Children with all types of verifiable SEN were eligible. *Inclusion* refers to an educational setting with a mixture of children with and without SEN. *Segregation* refers to the separate education of children with SEN. All studies that compared inclusive versus segregated educational settings for children with SEN were eligible. Qualitative studies were not included.

**Data Collection and Analysis:**

The total number of potentially relevant studies constituted 20,183 hits. A total of 94 studies met the inclusion criteria, all were non‐randomised studies. The 94 studies analysed data from 19 different countries. Only 15 studies could be used in the data synthesis. Seventy‐nine studies could not be used in the data synthesis as they were judged to be of critical risk of bias and, in accordance with the protocol, were excluded from the meta‐analysis on the basis that they would be more likely to mislead than inform. The 15 studies came from nine different countries. Separate meta‐analyses were conducted on conceptually distinct outcomes. All analyses were inverse variance weighted using random effects statistical models. Sensitivity analyses were performed to evaluate the robustness of pooled effect sizes across components of risk of bias.

**Main Results:**

The average baseline year of the interventions analysed in the 15 studies used for meta‐analysis was 2006, ranging from 1998 to 2012. The average number of participants analysed in the interventions was 151, ranging from 10 to 1357, and the average number of controls was 261, ranging from 5 to 2752. The studies included children with multiple types of disabilities such as learning disorders/intellectual disabilities, autism spectrum disorders, ADHD, physical handicaps, visual impairments, and Down syndrome. At most, the results from eight studies could be pooled in any of the meta‐analyses. All the meta‐analyses showed a weighted average that favoured the intervention group. None of them were statistically significant. The random effects weighted standardised mean difference was 0.20 (95% confidence interval [CI]: −0.01 to 0.42) for overall psychosocial adjustment; 0.04 (95% CI: −0.27 to 0.35) for language and literacy learning outcomes, and 0.05 (95% CI: −0.16 to 0.26) for math learning outcomes. There were no appreciable changes in the results as indicated by the sensitivity analyses. There was some inconsistency in the direction and magnitude of the effect sizes between the primary studies in all analyses and a moderate amount of heterogeneity. We attempted to investigate the heterogeneity by single factor sub‐group analyses, but results were inconclusive.

**Authors' Conclusions:**

The overall methodological quality of the included studies was low, and no experimental studies in which children were randomly assigned to intervention and control conditions were found. The 15 studies, which could be used in the data synthesis, were all, except for one, judged to be in serious risk of bias. Results of the meta‐analyses do not suggest on average any sizeable positive or negative effects of inclusion on children's academic achievement as measured by language, literacy, and math outcomes or on the overall psychosocial adjustment of children. The average point estimates favoured inclusion, though small and not statistically significant, heterogeneity was present in all analyses, and there was inconsistency in direction and magnitude of the effect sizes. This finding is similar to the results of previous meta‐analyses, which include studies published before 2000, and thus although the number of studies in the current meta‐analyses is limited, it can be concluded that it is very unlikely that inclusion in general increases or decreases learning and psychosocial adjustment in children with special needs. Future research should explore the effects of different kinds of inclusive education for children with different kinds of special needs, to expand the knowledge base on what works for whom.

## 1 PLAIN LANGUAGE SUMMARY

### 1.1 One size does not fit all—Inconsistent effects of inclusion on learning and psychosocial adjustment of children with special needs

The effects of placing children with special needs in grades K‐12 into inclusive educational settings are inconsistent. Findings from this review suggest that, in general, inclusion neither increases nor decreases learning and psychosocial adjustment of children with special needs in the OECD countries.

### 1.2 What is this review about?

This review and meta‐analysis summarises evidence from studies exploring the effects of inclusion on children with special needs in regular educational settings, compared with segregated educational settings.

The review summarises evidence from 15 non‐randomised studies in three meta‐analyses on language and literacy outcomes, math outcomes and overall psychosocial adjustment of children.

**What is the aim of this review?**

This review aims to explore the effects of placing children with special needs in inclusive educational settings, compared with segregated placement on measures of academic achievement and psychosocial adjustment. A secondary aim is to explore how different child or setting characteristics (gender, age, type and severity of special need, part‐ or full‐time inclusive education, and co‐teaching) might moderate outcomes.

### 1.3 What studies are included?

This review includes studies that evaluate the effects of placing children with special educational needs in mainstream educational settings (inclusion). A total of 94 studies were identified. However, only 15 of these were assessed to be of sufficient methodological quality to be included in the meta‐analyses and only between six and eight studies could be synthesised in each of the meta‐analyses.

Four studies were from the USA, three from the UK, two from the Netherlands, and one each from Switzerland, Finland, Germany, the Czech Republic, Belgium and Denmark.

The studies included children with multiple types of disabilities such as learning disorders/intellectual disabilities, autism spectrum disorders, ADHD, physical handicaps, visual impairments, and Down syndrome.

The studies all had important methodological weaknesses. None of the included studies used experimental designs with random assignment.

### 1.4 What are the main findings of this review?

Results of the meta‐analyses do not suggest any consistent positive or negative effects of inclusion on children's academic achievement as measured by language, literacy and math outcomes, or on the overall psychosocial adjustment of children. The studies in the analysis demonstrated a wide range of both large positive and large negative effect sizes; and although the average effect sizes did favour inclusion, they were small and none were statistically significant.

### 1.5 How has this intervention worked?

Supporters of mainstreaming or inclusion claim that segregated educational placement causes stigmatisation and social isolation, which may have detrimental effects on the self‐concept, social identity, and self‐confidence of students with special educational needs.

On the other hand, opponents suggest that placement in general education classrooms may have adverse effects for children with special needs, especially if the time and resources allocated for individualisation and differentiation are not aligned with student needs.

In line with these opposing positions, the findings from this review suggest that while some children with special needs may benefit from inclusive educational placement, other children may benefit from traditional special education in a segregated setting. Unfortunately, it was not possible to explore the effects of different kinds of inclusive education for different kinds of children with special needs.

### 1.6 What do the findings of this review mean?

The overall effects of inclusion on the academic achievement and overall psychosocial adjustment of students with special needs are inconsistent. Our findings are very similar to the results of previous systematic reviews and meta‐analyses, which include studies published before 2000. It is very unlikely that inclusion in general increases or decreases learning and psychosocial adjustment in children with special needs.

These findings point to the need for an individual assessment of the specific child's educational and psychosocial needs rather than a one‐size‐fits‐all approach to placement in special education.

Research should explore the effects of different kinds of inclusive education for different kinds of children with special needs, to expand the knowledge base on what works for whom.

### 1.7 How up‐to‐date is this review?

The review authors searched for studies up to 30 September 2021.

## 2 BACKGROUND

### 2.1 Description of the condition

The term *children with special educational needs* (SEN) refers to highly diverse populations of children with a wide range of physical, cognitive, and socioemotional disabilities or difficulties as well as strengths and resources causing them to require varying degrees of special educational support and assistance (Squires, 2012; Vehmas, 2010; Wilson, 2002).

Several studies document significant gender imbalances in the number of students who receive special educational support, and for most disability categories, the prevalence is higher for boys (Skårbrevik, 2002). The reasons for these imbalances are likely complex, and it is beyond the scope of the present review to account for the hypotheses and findings associated with each disability category. However, a general hypothesis across different categories of SEN is the notion that SEN are more likely to remain undetected in girls as symptoms and difficulties in girls may be less visible to educators (Arms et al., 2008).

Traditional special education consists of segregating students with special needs from mainstream students within separate and typically smaller classrooms or educational settings. However, as early as in the 1930s, a movement originally known as *mainstreaming*, and in more recent years as *inclusion*, has sought to bring an end to segregated placement as the preferred educational option for students with special needs (Carlberg & Kavale, 1980). In 1994, the idea of inclusive education became even more consolidated when the Salamanca Statement was adopted by representatives from 92 countries, resulting in an international shift in policy. This meant that far more students with special needs started entering general educational settings around the globe (Murawski & Lee Swanson, 2001; Ruijs & Peetsma, 2009).

The terms *inclusion, mainstreaming, integrated placement*, and *cross‐categorical instruction* all refer to educational settings with a group composition consisting of a mixture of students with and without SEN. In the present review, we have chosen to use the term *inclusion* to refer to general educational settings in which there is a mixture of students with and without SEN. Ideally, inclusion and inclusive education should be based on an educational approach in which the goal is to continuously address and respond to the diversity of needs of all learners through increasing participation and reducing exclusion within and from education. Inclusion thus may involve changes and modifications in content, approaches, structures, and strategies, with a common vision which covers all children and a conviction that it is the responsibility of the regular system to educate all children. Inclusion emphasises the provision of opportunities for equal participation of children with disabilities (physical, social, and/or emotional) whenever possible into general education, but leaves open the possibility of personal choice and options for special assistance and facilities for those who need it (UNESCO, 2019). Inclusion as an ideological and theoretical movement was built on a philosophical foundation, but during the last sixty years, the number of empirical studies addressing inclusive education has grown tremendously. However, findings on the efficacy of inclusion on student outcomes are still far from unequivocal (Kavale & Forness, 2000; Lindsay, 2007; Ruijs & Peetsma, 2009). This is where the present review contributes, as the aim of the review was to summarise contemporary evidence on the effects of inclusive education when compared to a traditional segregated approach on students' learning, socioemotional adjustment, and well‐being. It is important to consider the types of students who might benefit most from inclusive education. As stated earlier, it is possible that the effects of inclusive education may be different for girls and boys. Further, a child's cognitive and socioemotional skills and needs develop throughout childhood and adolescence (Lightfoot et al., 2009) and thus, it is possible that the potential benefits of inclusive education may vary depending on age and other characteristics of the child.

### 2.2 Description of the intervention

At the core of inclusion is the principle that students with special or additional learning needs or disabilities belong in mainstream education. The fundamental principle of inclusive education is that all children should learn together, regardless of any difficulties or differences (UNESCO, 2019; Winter & O'Raw, 2010). However, operationally within the present review, we define *inclusion* as an educational setting with a mixture of children with and without SEN. In the present review, the intervention termed *inclusion* may thus be defined as any given group composition within a general educational setting which contains at least one child with an identified SEN.

Based on the core principles of inclusion, there are many ways in which inclusion may be practised and adjusted, and thus there are numerous characteristics within the inclusive setting, which may vary across the included studies. This review included studies of all kinds of inclusive education meaning that placement in the inclusive setting may be full time or part‐time. Special education students are a diverse group, as outlined in the section entitled ‘Types of participants’, and studies were included regardless of the type of SEN of the student population and regardless of the ratio of students with and without special needs within the inclusive setting. We included studies in which the general education teachers are provided with support and continuing professional development aimed at helping the teachers accommodate the needs of special education students and studies of inclusive settings in which no such support was offered to the teachers. It is often referred to as co‐teaching when two or more professionals deliver substantive instruction to a diverse or blended group of students within the same physical space (Murawski & Lee Swanson, 2001). In the present review, we also included studies in which special education teachers and/or teaching assistants were present within the general education setting (co‐teaching) and studies in which they were not.

This list of possible variations in student and classroom characteristics is not exhaustive, and in summary, within the present review, we included studies of all kinds of inclusive education as long as the studies were aimed at exploring the effects of inclusion in comparison to segregated special educational settings. We planned to conduct moderator analyses to explore the impact of specific characteristics of the inclusive educational setting and the characteristics of SEN on student outcomes.

### 2.3 How the intervention might work

Considering the rapid global development towards inclusive educational placement for students with SEN, there is a rather surprising lack of pedagogical, psychological, or didactic theories regarding the specific ways in which inclusive education may affect students with special needs' academic and socioemotional development. Group composition within the educational setting may play a significant role in determining the academic achievement, socioemotional development, and overall wellbeing of students with special needs. Theoretically and ideologically, scholars favouring mainstreaming or inclusion propose that segregated educational placement causes stigmatisation and social isolation which may have detrimental effects on the self‐concept, social identity, and self‐confidence of students with SEN (Dyssegaard & Larsen, 2013). Secondly, being placed in a general education classroom along with typically developing peers is proposed to benefit students' academic growth through peer effects (Rea et al., 2002; Tremblay, 2013). Finally, it is hypothesised that social interaction with general education peers may provide developmental opportunities that are not present in smaller, specialised units (Fisher & Meyer, 2002).

On the other hand, opponents of inclusive education suggest that placement in general education classrooms may have adverse effects for students with special needs, especially if the time and resources allocated for individualisation and differentiation are not aligned with student needs. In such cases, students' learning opportunities and wellbeing may also suffer, resulting in damages to self‐concept (Zeleke, 2004), social isolation or bullying (Monchy et al., 2004; Pijl et al., 2010), stress (Pitt & Curtin, 2004), negative self‐perception, and lower self‐confidence (Bakker et al., 2007; Ruijs & Peetsma, 2009).

Hegarty (1993) provides a narrative review of the literature on inclusion and suggests that a number of factors are associated with positive student outcomes in inclusive settings. These are: (1) instruction based on student achievement needs, (2) materials and procedures that allow students to proceed at their own pace, (3) additional time for students who need it, (4) increased student responsibility for their own learning, (5) co‐operation among students in achieving goals, (6) support teaching, and (7) collaboration among special and general education teachers.

In sum, the impact of inclusion on the academic achievement, socioemotional development, and wellbeing of students with special needs may be hypothesised to be both positive and negative, and the current knowledge base is rather unclear, leaving special educators and policymakers uncertain when making decisions on special education provision.

### 2.4 Why it is important to do this review

Since the 1980s, a number of reviews on the impact of inclusion on students with special needs have been published (Hegarty, 1993; Madden & Slavin, 1983; Ottenbacher & Cooper, 1982; Wang & Baker, 1985). Results are equivocal, and several reviews point to a number of methodological challenges and weaknesses of the study designs within the included primary studies. In summary, most reviews suggest a neutral or small positive impact of inclusion on most outcomes. However, all reviews also point to the need to study the impact of potential moderators more thoroughly, as there may be several interaction effects between student and classroom characteristics, such *as student disability category* × *proportion of students with disabilities within the classroom* and *disability category* × *presence of teaching assistants*. Therefore, it was important to conduct the present review to explore the impact of potential moderators associated with student and classroom characteristics.

In the following section, we present the existing reviews and their main findings.

In a systematic review and meta‐analysis, which included 50 primary studies exploring the effects of special versus regular class placement for children with special needs, Carlberg and Kavale (1980) concluded that for students with special needs consisting of below average IQs, special classes were significantly inferior to regular classes on all outcome measures (separate analyses were carried out for achievement, social/personal, and other measures). However, for students with behavioural disorders, emotional disturbances, and learning disabilities, special classes were superior to regular classes.

Madden and Slavin (1983) conducted a narrative review of the effects of mainstreaming/inclusion on students with mild academic disabilities. The review does not include a description of the search strategy for identifying records or the criteria used to determine eligibility for inclusion in the review. The review concludes that among methodologically adequate studies, findings indicate few benefits on academic and social outcomes of placement in full‐time special education compared with part‐time placement with resource support or full time regular class placement for students with mild academic disabilities.

Ottenbacher and Cooper (1982) conducted a systematic review and meta‐analysis, which included 43 primary studies exploring the effects of class placement (special class, regular class and resource class defined as placement in regular education classroom with resource support and the possibility for part‐time segregated education) on the social adjustment of students with mild cognitive disabilities. The overall results suggest a very small effect in favour of special class placement over regular class placement. However, when special class placement was compared with resource class placement, results were insignificant but favouring resource class placement.

Wang and Baker (1985) conducted a systematic review and meta‐analysis, which included 11 primary studies exploring the effects of mainstreaming/inclusion on children with SEN. To be eligible for inclusion in this review, primary studies needed to provide information on the effects of mainstreaming on students with special needs placed in a regular education setting. The studies had to use a control group consisting of special needs students with comparable impairment classifications placed in a segregated learning environment. The final selection of studies included 11 studies published between 1975 and 1984. The included studies used a wide variety of outcomes, but within the meta‐analysis, outcomes were synthesised into three categories: performance, attitudinal, and process effects, and separate analyses were carried out for each of the three outcome categories. The study found small‐to‐moderate beneficial effects of inclusion on all outcomes with an overall mean weighted effect size across all studies and all three categories of outcomes of 0.33.

Hegarty (1993) provides a narrative review of the literature on integration (inclusion) of students with different disabilities. The narrative review is based on a literature review which was commissioned by the Centre for Educational Research and Innovation under the Organisation for Economic Cooperation and Development (OECD) and was conducted by researchers in five different countries. The review does not include a description of the search strategies for identifying records or the criteria used to determine eligibility for the selected primary studies. Within the review, a number of factors which are associated with effective integration programmes are identified.

Baker et al. (1995) describe a review and meta‐analysis by Baker et al. (1995), which included 13 primary studies exploring the effects of inclusive placement on academic and social outcomes for students with special needs. We have been unable to retrieve the original publication, but according to Baker et al. (1995), this study found a very small effect in favour of inclusive placement on academic outcomes (0.08) and a small to moderate effect on social outcomes (0.28).

Sebba and Sachdev (1997) provide a review as part of a research report on what works for whom in inclusive education. The review does not include a description of the search strategy or the criteria for inclusion/exclusion of studies for the review. Within the research report, the authors suggest an overall positive impact of inclusive education and list a number of potential moderators such as attitudes of teachers and parents as well as a number of recommendations for the implementation of inclusive education.

McGregor and Vogelsberg (1998) provide a narrative review of studies of both the effects of inclusive schooling on student outcomes and studies focusing on issues related to the implementation of inclusion. The review includes both quantitative and qualitative studies including case studies. Results are difficult to synthesise, but suggest an overall positive impact of inclusion based on the main findings: (1) students with disabilities demonstrate high levels of social interaction in settings with typically developing peers, but placement alone does not guarantee positive social outcomes; (2) interactive small group contexts facilitate skill acquisition and social acceptance; (3) friendships develop between students with disabilities and typically developing peers.

Freeman and Alkin (2000) conducted a systematic narrative review in which it was concluded that on measures of academic achievement and social competence, children with mental retardation placed in general education perform better than children with mental retardation placed in special education classrooms. This review was only about children with mental retardation and did not include meta‐analyses.

Murawski and Lee Swanson (2001) conducted a systematic review and meta‐analysis which included 6 studies exploring the effectiveness of co‐teaching on student outcomes of both general education students and students with SEN. Co‐teaching was defined as two or more professionals delivering substantive instruction to a diverse or blended group of students within a shared/common physical space, and thus in this review co‐teaching is a form of inclusion. The outcomes within the included studies were grades, achievement scores, social, and attitudinal outcomes. The review found co‐teaching to be effective (average total effect size of 0.40). It is unclear what the control conditions within the included studies were and two of the included studies did not have a control group, but used a pre‐test/post‐test research design.

Lindsay (2007) provides a narrative review of the effectiveness of inclusive education for students with SEN. The review provides a historical overview of the vast literature before 2000 and a search of studies published 2001–2005 in eight journals on special education. The search identified 1373 studies and points to the fact that only 1% of the identified papers were comparative outcome studies. The review concludes that there is a lack of evidence for the effectiveness of inclusion and argues that where evidence does exist, the balance is only marginally positive. Lindsay (2007) thus supports the need for an updated systematic review and meta‐analysis on the effectiveness of inclusion for students with special needs, with special attention to the potential impact of student and classroom moderators.

In a systematic narrative review of the effects of inclusion on both learning and socioemotional outcomes of students with and without special needs, Ruijs and Peetsma (2009) point to mixed findings regarding the effects of inclusion on student outcomes and suggest a number of potential moderators. The authors conclude that there is a need for more research. This review has not been updated since publication and doesn't include meta‐analyses.

In 2009, a systematic review of evidence comparing the academic performance of students with special needs in different educational settings was carried out by the Canadian Council on Learning. The review included 30 primary studies. The search strategy for identifying studies was not described. The included studies examined students with learning disabilities, intellectual disabilities, language impairments, and mixed disabilities. The quality of each study was rated as either ‘high’, ‘medium’ or ‘low’ based on criteria related to transparency and research design, and effect sizes were retrieved. No meta‐analyses were carried out, but the authors provide tables illustrating the number of effect sizes for each disability category favouring either inclusive or segregated settings along with the quality ratings of the studies from which they were retrieved. The authors point to mixed findings, but conclude that the balance of evidence shows favourable academic outcomes for students with SEN educated in inclusive settings, however they also note that results are not homogenous, and that effects are generally small in magnitude.^1^

Dyssegaard and Larsen (2013) provide a systematic review and narrative synthesis on the effects of including children with special needs in mainstream teaching in primary and lower secondary school, and on which of the applied educational methods have proven to have a positive effect. The narrative synthesis is based on 43 studies of which 16 studies were deemed to have a ‘high level of evidence’. The included studies consist of randomised controlled trials, non‐randomised controlled trials, systematic reviews, cohort studies, longitudinal studies, and studies using a pre‐test/post‐test design. The systematic review included studies focusing on outcomes for both mainstream and special needs students and does not include a meta‐analysis. The conclusion points to mixed findings regarding the overall effectiveness of inclusion on the academic achievement and psychosocial adjustment of special needs students and suggests that the effects may vary depending on the age of the child and the overall school and teacher attitudes towards inclusion. Furthermore, the review suggests that the effectiveness of co‐teaching may depend on the educational background and continuous professional development of both special and general education teachers and of teaching assistants.

Carrol et al. (2017) provide a rapid evidence assessment of studies focused on approaches, strategies, and interventions supporting children and young people with SEN in mainstream schools. The rapid evidence assessment is based on a systematic search in a single database (ERIC) as well as a strategy of consulting experts within the relevant fields. The initial search identified 1046 papers of which 505 were later excluded due to low quality of evidence. The rapid evidence assessment points to a number of implementation strategies, pedagogical, and didactic approaches which have shown positive results. Furthermore, the study points to evidence gaps and suggests the need for further research. The rapid evidence assessment does not include a meta‐analysis.

In the present review, besides being up‐to‐date, we conducted an extensive risk of bias assessment of all included studies, and we provide separate meta‐analyses for each conceptual outcome (academic achievement: language/literacy outcomes and math outcomes, and psychosocial adjustment). Furthermore, we hoped to be able to conduct moderator analyses based on the children's specific disability categories and the specific type of inclusion setting.

Traditional segregated special education is costly and in a time of increased interaction between special and general education systems and constraints on educational spending, policymakers must consider the cost‐efficiency of different special needs provisions.

As more students with SEN enter general education settings, educators and policymakers must consider how the needs of these students are met in different settings and on what grounds placement in general or special educational settings should be determined. As previously noted, the current knowledge base is ambiguous with many findings suggesting a complex interplay between student and classroom characteristics (Carlberg & Kavale, 1980; Mesibov & Shea, 1996; Peetsma et al., 2001), leaving special educators and policymakers uncertain when making decisions on special education provision and highlighting the need for a comprehensive review of the effectiveness of *inclusion* on student outcomes.

## 3 OBJECTIVES

The objective of this systematic review was first:

- To uncover and synthesise data from studies to assess the effects of inclusion on measures of academic achievement, socioemotional development, and wellbeing of children with special needs when compared to children with special needs who receive special education in a segregated setting.
- A secondary objective was to explore how potential moderators (gender, age, type and severity of special need, part‐ or full‐time inclusive education, and co‐teaching) affect the outcomes.

## 4 METHODS

### 4.1 Criteria for considering studies for this review

#### 4.1.1 Types of studies

To summarise what is known about the causal effects of inclusion on Student's academic achievement, socioemotional outcomes, and wellbeing in special education, we included all studies with a well‐defined control group. Thus, the study designs eligible for inclusion were:

A. Randomised and quasi‐randomised controlled trials (allocated at either the individual level or cluster level, e.g., class/school/geographical area etc.).
B. Non‐randomised studies (inclusion has occurred in the course of usual decisions, the allocation to inclusive and segregated special educational placement is not controlled by the researcher, and there is a comparison of two or more groups of participants, i.e., at least a treated group and a control group).

Studies using a single group pre‐test/post‐test research design were not eligible for inclusion in the review. Non‐randomised studies using an instrumental variable approach were not eligible.

To minimise the risk of bias in cluster‐randomised studies, we excluded study designs in which only one unit was assigned to the intervention or control group. That is, there had to be at least two units in the intervention group and two units in the control group for the study to be eligible, as there is otherwise a substantial risk of confounding treatment effects with ‘unit’ effects (in this case, ‘unit’ would likely be school).

To maximise the relevance of findings from the present review to current policy and decision‐makers, we limited our search to studies published after 2000. The reason for excluding older studies was twofold. First, as described previously, a number of systematic reviews and meta‐analyses have already synthesised the effects of inclusion based on studies published before 2000. Second, educational settings, pedagogical approaches, and the development and availability of technological tools to support the educational needs of children have undergone major changes throughout the past two decades (Cheng & Lai, 2019), and in order for the findings from the present review to be applicable to the current realities within educational settings, we limited our review to the more recent findings.

#### 4.1.2 Types of participants

##### Types of participants

The review included studies of children with special needs in grades K‐12 (or the equivalent in European countries) in special education in the Western world defined as the OECD countries. The reasons for focusing on the OECD countries are twofold; first, we believe that the way in which children with disabilities are perceived within society is culturally embedded, which creates fundamental differences in the life circumstances for children living with disabilities around the globe (Maloni et al., 2010; McNally & Mannan, 2013). Second, special education is costly and thus the resources available for providing special educational support for children with special needs are often fundamentally different between countries in the OECD and the developing countries (Sibanda, 2018; UNESCO, 2019).

Some controversy exists regarding the definition of what constitutes a SEN (Vehmas, 2010; Wilson, 2002). A widely used definition can be found in the US Individuals with Disabilities Education Act (IDEA), in which special needs are divided into 13 different disability categories under which children are eligible for services.^2^ These categories are:

- specific learning disability (covers challenges related to a child's ability to read, write, listen, speak or do math, e.g., dyslexia or dyscalculia),
- other health impairment covers conditions limiting a child's strength, energy, or alertness, for example, ADHD,
- autism spectrum disorder (ASD),
- emotional disturbance (may include, e.g., anxiety, obsessive‐compulsive disorder and depression),
- speech or language impairment (covers difficulties with speech or language, e.g., language problems affecting a child's ability to understand words or express herself),
- visual impairment (covers eyesight problems, including partial sight and blindness),
- deafness (covers instances where a child cannot hear most or all sounds, even with a hearing aid),
- hearing impairment (refers to a hearing loss not covered by the definition of deafness),
- deaf‐blindness (covers children suffering from both severe hearing and vision loss),
- orthopaedic impairment (covers instances when a child has problems with bodily function or ability, as in the case of cerebral palsy),
- intellectual disability (covers below‐average intellectual ability),
- traumatic brain injury (covers brain injuries caused by accidents or other kinds of physical force),
- multiple disabilities (children with more than one condition covered by the IDEA criteria).

However, the above listed criteria are not to be conceived as exhaustive or as clear‐cut definitions of what constitutes SEN, but are rather seen as guidance tools in the search for and screening of relevant studies. We acknowledge that existing attempts to define SEN, as discussed in Vehmas (2010) and Wilson (2002), are characterised by a lack of clarity, which requires us to be transparent as to our own use of the term throughout the review process. For the purpose of this review, we included studies of all types of verifiable special needs, that is children who receive special educational support and/or who have been diagnosed with any kind of disability.

#### 4.1.3 Types of interventions

*Inclusion* refers to an educational setting with a mixture of children with and without SEN. In the present review, the intervention termed *inclusion* may thus be defined as any given group composition within a general educational setting which contains at least one child with an identified SEN. Within some studies, inclusion may also be referred to as *integration, mainstreaming*, *integrated placement*, and *co‐teaching with a blended student population*.

Inclusion may be full‐time or part‐time and may involve additional teaching and/or pedagogical resources. We included studies of all kinds of inclusive education.

We excluded children in home‐ or preschool and children in residential schools.

#### 4.1.4 Types of outcome measures

##### Types of outcome measures

In the present review, we aimed to extract the following outcomes.

###### Academic Achievement

Academic achievement outcomes include reading and mathematics as well as measures of other academic subjects and global academic performance. Outcome measures must be standardised measures of academic achievement such as standardised literacy tests (e.g., reading, spelling, and writing) and standardised numeracy tests (e.g., mathematical problem‐solving, arithmetic and numerical reasoning, grade level math), standardised tests in other academic subjects (e.g., in science or second language).

###### Socioemotional outcomes

Socioemotional outcomes refer to validated measures of children's psychological, emotional and social adjustment, and mental health.

###### Wellbeing

Wellbeing refers to measures of children's subjective quality of life, self‐perception, self‐esteem, and self‐image.

Effects favouring the control conditions were also extracted and reported.

Studies who do not report on any of the outcomes listed above were excluded from the review.

#### 4.1.5 Primary outcomes

Academic achievement, socioemotional outcomes, and wellbeing were all primary outcomes.

#### 4.1.6 Secondary outcomes

School completion rates were secondary outcomes.

##### Duration of follow‐up

We included post‐intervention outcomes measured during and after placement in an inclusive educational setting. None of the included studies had follow‐up measures which could be used in the meta‐analysis.

### 4.2 Search methods for identification of studies

#### 4.2.1 Search strategy

Relevant studies were identified through electronic searches of bibliographic databases, governmental and grey literature repositories, hand search in specific targeted journals, attempts to contact experts, and Internet search engines. The electronic database searches were completed on 24 April 2021 and other resources were searched in August and September 2021. We searched to identify both published and unpublished literature. The searches were international in scope. The searches were limited to publications published from 2000 onward to maximise contemporary relevance of the review. Reference lists of included studies used in the meta‐analysis were also searched.

We implemented a wide range of search methods and strategies to maximise coverage of relevant references, while simultaneously attempting to reduce different types of bias related to publication and dissemination systems.

Subject terms in the facets were selected according to the thesaurus or index of each database. Keywords were supplied if the search technique provided additional results. Use of truncation and wildcards were used to address English spelling variants.

The different strategies and methods are presented below and detailed documentation of the searches is available in Supporting Information: Appendices 1, 2, and 4.

#### 4.2.2 Electronic searches

The following electronic databases were searched:

- Academic Search Premier (EBSCO) (1953–)
- APA PsycINFO (EBSCO) (1846–)
- EconLit (EBSCO) (1969–)
- ERIC (EBSCO)(1915–)
- International Bibliography of the Social Sciences (ProQuest) (1951–)
- Sociological Abstracts (ProQuest) (1952–)
- Science Citation Index Expanded (Web Of Science) (1900–)
- Social Sciences Citation Index (Web Of Science) (1956–)
- SocINDEX (EBSCO) (1908–)

Details can be found in Supporting Information: Appendices 1 and 2.

#### 4.2.3 Example of a search string

Below is an example of a search‐string utilised to search PsycINFO through the EBSCO‐platform. This search string was modified according to the search interface, syntax, and subject terms for each of the above standing databases.

**Table:** 

Search	Search terms	Results
S13	S4 AND S8 AND S12	3182
S12	S9 OR S10 OR S11	2,830,386
S11	DE ‘Effect Size’ OR DE ‘Control Groups’ OR DE ‘Experimental Groups’ OR DE ‘Experiments’ OR DE ‘Matched Groups’ OR DE ‘Quasiexperimental Design’ OR DE ‘Randomized Controlled Trials’ OR DE ‘Comparative Testing’	1235
S10	AB (effect* OR trial* OR experiment* OR control* OR random* OR impact* OR compar* OR difference*)	2,696,492
S9	TI (effect* OR trial* OR experiment* OR control* OR random* OR impact* OR compar* OR difference*)	739,679
S8	S5 OR S6 OR S7	352,254
S7	DE ‘Placement’ OR DE ‘Academic Accommodations (Disabilities)’ OR DE ‘Inclusion’ OR DE ‘Mainstreaming’ OR DE ‘Student Placement’	965
S6	AB (integrat* OR immers* OR inclus* OR mainstream* OR placement*)	336,471
S5	TI (integrat* OR immers* OR inclus* OR mainstream* OR placement*)	61,274
S4	(S1 AND S2) OR S3	39,663
S3	DE (‘Special Needs Students’)	51,020
S2	TI (need* OR special* OR additional*) OR AB ((special* OR additional* OR educational*) N5 (need*))	94,319
S1	TI (student* OR pupil* OR child* OR youth* OR young*) OR AB (student* OR pupil* OR child* OR youth* OR young*)	1,371,893

##### Description and rationale for search terms and facets, and sensitivity of the search string

The search string was designed to balance sensitivity and precision. The search string contains three aspects related to the inclusion criteria of the review. To keep the search string sufficiently sensitive, we searched each aspect in either title, abstract, or subject terms.

- Search 1–4 covers the population
- Search 5–8 covers the intervention
- Search 9–12 covers the study type/methodology
- Search 13 combines the three aspects

##### Limitations of the search‐string

Searches were limited to from 01/01/2000 and forwards to maximise contemporary relevance of findings. We did not implement any language restrictions to our search, but we only included studies in English, Danish, Norwegian, and Swedish.

#### 4.2.4 Searching other resources

##### Searching other resources

We searched a range of web‐based resources in August and September 2021 to identify references that were either unpublished, not in English, or both.

Due to the language restrictions of the review team, we selected Danish, Swedish, and Norwegian as ‘other languages’ to search in, to identify relevant unpublished literature.

Some of the resources listed contain multiple types of unpublished literature, as well as published references. The resources we searched are listed under the category of literature that is most prevalent in the resource. Searches were carried out by combining all meaningful relevant key terms such as: special needs students, placement, achievement, students with disabilities, additional education, special education, mainstreaming, inclusion, co‐teaching. When performing searches in Google and Google scholar the screening of records was discontinued, when it was deemed unlikely to result in relevant results. A detailed description of each search is available in Supporting Information: Appendix 4, in which the number of records screened based on each search is also documented.

*Searches for working papers and conference proceedings in English*

- American Educational Research Association (AERA)—https://www.aera.net/
- European Educational Research Association (EERA)—https://eera-ecer.de/
- NBER working paper series—http://www.nber.org
- Open Grey—http://www.opengrey.eu/
- OECD iLibrary—https://www.oecd-ilibrary.org/
- Social Science Research Network—https://www.ssrn.com/index.cfm/en/

*Searches for dissertations and theses in English*

- EBSCO Open Dissertations (through EBSCO interface)
- ProQuest Dissertation and Theses (through ProQuest interface)

*Searches for reports in English*

- Google Scholar—https://scholar.google.com/

*Searches for ongoing studies in English*

- Best Evidence Encyclopedia—http://www.bestevidence.org/
- Google searches—https://www.google.com/
- Social Care Online—https://www.scie-socialcareonline.org.uk/

*Searches for working papers, conference proceedings, dissertations and theses in other languages*

- AAU Publications—Academic publications from the University of Aarhus https://pure.au.dk/portal/da/organisations/8000/publications.html
- DIVA – Swedish Digital Scientific Archives—http://www.diva-portal.org/smash/
- Forskning.ku—Academic publications from the university of Copenhagen ‐ https://forskning.ku.dk/soeg/
- NORA ‐ Norwegian Open Research Archives—http://nora.openaccess.no/
- Skolporten—Swedish Dissertations—https://www.skolporten.se/forskning/
- SwePub—Academic publications at Swedish universities—http://swepub.kb.se/

*Searches for reports in other languages*

- Google Scholar—https://scholar.google.com/
- Google searches—https://www.google.com

We did not search for government documents separately as the likelihood of finding additional eligible studies was deemed to be too low.

##### Hand searches

We implemented hand searches in key journals to identify references that were poorly indexed in the bibliographical databases, as well as covering references that were published in a journal, but not yet indexed in the bibliographical databases during the search process.

Our selection of journals to hand search was based on the frequency of the journals in our pilot searches for designing the search strings in the protocol phase. Journals with the highest frequency of references in the pilot searches were selected for hand search. Hand searches were carried out in all journals for the years 2019, 2020, and 2021. We searched the following journals:

- *British Journal of Educational Psychology*
- *British Journal of Special Education (BJSE)*
- *Disability Studies Quarterly*
- *Disability, Development and Education*
- *European Journal of Special Needs Education*
- *Exceptional Children*
- *Intellectual and Developmental Disabilities*
- *International Journal of Educational Management*
- *International Journal of Inclusive Education*
- *Journal of Intellectual Disability Research*
- *Journal of Learning Disabilities*
- *Journal of Research in Special Educational Needs (JORSEN)*
- *Mental Retardation*
- Teacher Education and Special Education

##### Search for systematic reviews

Before writing the protocol for this review, we developed a specific search string to identify other systematic reviews in the databases listed above. This was done simultaneously with the development of the search string described above, and the identified relevant reviews are considered in this review.

We also searched for further systematic reviews on the following resources in September 2021:

- Campbell Journal of Systematic Reviews—https://campbellcollaboration.org/
- Cochrane Library—https://www.cochranelibrary.com/
- Centre for Reviews and Dissemination Databases—https://www.crd.york.ac.uk/CRDWeb/
- EPPI‐Centre database of education research—https://eppi.ioe.ac.uk/webdatabases/Intro.aspx?ID=6

Detailed descriptions of searches are available in the Supporting Information Appendices.

##### Checking reference lists of included studies

We checked the reference lists for the 15 studies used in the meta‐analysis.

##### Contact to experts

We attemped to contact a small number of experts to identify unpublished and ongoing studies, and provided them with the inclusion criteria for the review along with the list of included studies, asking for any other published, unpublished, or ongoing studies relevant for the review. None of these contacts lead to the inclusion of additional studies for the review.

### 4.3 Data collection and analysis

The total number of potential relevant studies constituted 20,183 hits after duplicates were removed. A total of 94 studies met the inclusion criteria and were critically appraised by the review authors. The 94 studies analysed data from 87 different samples. Only 15 studies (analysing 15 different samples) could be used in the data synthesis. 79 studies could not be used in the data synthesis, as they were judged to have too high risk of bias and, in accordance with the protocol, were excluded from the meta‐analysis on the basis that they would be more likely to mislead than inform. One study (Rangvid, 2021) did not have any outcomes in common with the other 14 studies and could therefore not be synthesised in a meta‐analysis but was reported in an additional table.

Meta‐analyses of three child outcomes (overall psychosocial adjustment, language/literacy learning outcomes, and math learning outcomes) were conducted on each metric separately. All analyses were inverse variance weighted using random effects statistical models that incorporate both the sampling variance and between study variance components into the study level weights. Random effects weighted mean effect sizes were calculated using 95% confidence intervals (CIs).

A number of other relevant outcomes were reported in a single study only (measures on academic tests other than language/literacy and math, learning motivation, parent and teacher reported SDQ on friendship and bullying, socialisation skills and school completion rates) and could therefore not be synthesised in the meta‐analyses. The effect sizes and 95% CIs were calculated and reported in an additional table.

#### 4.3.1 Selection of studies

In the present review, we included studies of children with special needs in grades K‐12 (or the equivalent in European countries) in special education in the Western world defined as the OECD countries. Special needs were defined broadly, and the review included studies of all types of verifiable special needs, that is, children who receive special educational support and/or who have been diagnosed with any kind of disability. The intervention of interest in the present review is inclusion. Inclusion refers to an educational setting with a mixture of children with and without SEN. In the present review, inclusion was thus defined as any given group composition within a general educational setting which contains at least one child with an identified SEN. Within some studies, inclusion may also be referred to as *integration, mainstreaming, integrated placement*, and *co‐teaching* with a blended student population. Inclusion may be full‐time or part‐time and may involve additional teaching and/or pedagogical resources. We included studies of all kinds of inclusive education. We excluded studies of children in home‐ or preschool and children in residential schools.

All study designs that used a well‐defined control group were eligible for inclusion. Studies that utilised qualitative approaches were not included.

Under the supervision of review authors, two review team assistants independently screened titles and abstracts to exclude studies that were clearly irrelevant. Studies considered eligible by at least one assistant or studies where there was insufficient information in the title and abstract to judge eligibility, were retrieved in full text. The full texts were then screened independently by two review team assistants under the supervision of the review authors. Any disagreement of eligibility was resolved by the review authors by discussion.

#### 4.3.2 Data extraction and management

Two review authors independently coded and extracted data from included studies. A coding sheet was piloted on several studies and revised as necessary. Disagreements were resolved by consulting a third review author with extensive content and methods expertise. Disagreements were minor and were resolved by discussion. Data and information was extracted on: available characteristics of participants, intervention characteristics and control conditions, research design, sample size, risk of bias and potential confounding factors, outcomes, and results. Extracted data was stored electronically.

#### 4.3.3 Assessment of risk of bias in included studies

##### Assessment of risk of bias in included studies

We planned to use the ROB2 tool with included RCTs, but no RCT's were identified for inclusion in the review.

We assessed the risk of bias in the included non‐randomised studies, using the model ROBINS–I, developed by members of the Cochrane Bias Methods Group and the Cochrane Non‐Randomised Studies Methods Group (Sterne et al., 2016). We used the latest template for completion (currently it is the version of 19 September 2016).

The ROBINS‐I tool is based on the Cochrane RoB tool for randomised trials, which was launched in 2008 and modified in 2011 (Higgins et al., 2011).

The ROBINS‐I tool covers seven domains (each with a set of signalling questions to be answered for a specific outcome) through which bias might be introduced into non‐randomised studies:

(1) bias due to confounding;
(2) bias in selection of participants;
(3) bias in classification of interventions;
(4) bias due to deviations from intended interventions;
(5) bias due to missing outcome data;
(6) bias in measurement of the outcome;
(7) bias in selection of the reported result.

The first two domains address issues before the start of the interventions and the third domain addresses classification of the interventions themselves. The last four domains address issues after the start of interventions and there is substantial overlap for these four domains between bias in randomised studies and bias in non‐randomised studies (although signalling questions are somewhat different in several places, see Sterne et al., 2016, and Higgins et al., 2019).

Randomised study outcomes are rated on a ‘Low/Some concerns/High’ scale on each domain; whereas non‐randomised study outcomes are rated on a ‘Low/Moderate/Serious/Critical/No Information’ scale on each domain. The level ‘Critical’ means: the study (outcome) is too problematic in this domain to provide any useful evidence on the effects of intervention, and it is excluded from the data synthesis. The same critical level of risk of bias (excluding the result from the data synthesis) is not directly present in the RoB 2 tool, according to the guidance to the tool (Higgins et al., 2019).

We stopped the assessment of non‐randomised study outcomes as soon as one domain in the ROBINS‐I was judged as ‘Critical’.

‘Serious’ risk of bias in multiple domains in the ROBINS‐I assessment tool may also lead to a decision of an overall judgement of ‘Critical’ risk of bias for that outcome and when this occurred, the outcome or the study was excluded from the data synthesis.

##### Confounding

An important part of the risk of bias assessment of non‐randomised studies is consideration of how the studies deal with confounding factors. Systematic baseline differences between groups can compromise comparability between groups. Baseline differences can be observable (e.g., age and gender) and unobservable (to the researcher; e.g., children's motivation and ‘ability’). There is no single non‐randomised study design that always solves the selection problem. Different designs represent different approaches to dealing with selection problems under different assumptions, and consequently require different types of data. There can be particularly great variations in how different designs deal with selection on unobservables. The ‘adequate’ method depends on the model generating participation, that is, assumptions about the nature of the process by which participants are selected into a programme.

A major difficulty in estimating causal effects of inclusive education is the heterogeneity of children with SEN. In addition to the pre‐specified confounding factors, there may be unobservable factors affecting child development and wellbeing or invisible selection mechanisms causing certain types of families to choose a specific educational setting for their child for reasons unavailable to the researcher.

As there is no universally correct way to construct counterfactuals for non‐randomised designs, we looked for evidence that identification was achieved, and that the authors of the primary studies justified their choice of method in a convincing manner by discussing the assumption(s) leading to identification (the assumption(s) that make it possible to identify the counterfactual). Preferably, the authors should make an effort to justify their choice of method and convince the reader that students in inclusive versus segregated settings were comparable.

In addition to unobservables, we have identified the following observable confounding factors to be most relevant: performance at baseline, age/gender of the child, special needs category and impairment level, and socioeconomic background of the child's family. In each study, we assessed whether these factors were considered, and in addition we assessed if other factors were likely to be a source of confounding within the individual included studies.

##### Importance of pre‐specified confounding factors

The motivation for focusing on performance at baseline, age/gender of the child, special needs category and impairment level, and the socioeconomic background of the child's family is given below.

Performance at baseline is perhaps the most important potential confounding factor, as students with special needs constitute a highly diverse population. Thus, we looked for evidence that students in both intervention and control groups had similar academic performance at baseline.

The younger the child, the more dependent the child is on stimulating adult/child interaction. Therefore, the impact of inclusive versus segregated special education may vary depending on the age of the children, with younger children perhaps benefiting more from placement in smaller specialised units with a lower student/teacher ratio, meaning a lower number of students per teacher. Furthermore, puberty may bring about additional challenges for special education students, which may make them more socially and psychologically vulnerable to the stigma associated with having a SEN, and it is unclear if the potential social and psychological vulnerability is best handled within a general or special educational setting. In any case, it is highly possible that the effects of inclusion may vary depending on the age of the child.

From a very early age, gender is associated with differences in child behaviour and cognition (Chaplin & Aldao, 2013; Ostrov & Keating, 2004; Silverman, 2003). Little girls and boys often show different toy and play preferences (Todd et al., 2017), and a number of studies suggest that for diagnoses such as autism spectrum disorders and ADHD there are significant gender differences in the behavioural expressions of symptoms (Halladay et al., 2015), and thus it is possible that gender may have an impact on what constitutes the best educational setting for students with special needs.

As can be seen in the definition of SEN, the disability categories cover a very broad range of disabilities and there may be considerable variance in the impairment levels of students between the different disability categories. In the existing reviews, some results suggest that the effects of inclusive versus segregated placement may vary depending on the particular special education student population (see for instance Carlberg & Kavale, 1980; or the 2009 review from the Canadian Council of Education), which is why we consider this an important potential confounder.

A large body of research documents the impact of parental socioeconomic background on almost all aspects of children's development (Renninger et al., 2006), which is why we also consider it important to control for this.

##### Effect of primary interest and important co‐interventions

We were mainly interested in the effect of starting and adhering to the intended intervention, that is, the treatment on the treated (TOT) effect for students enroled in and attending inclusive education. The risk of bias assessments were therefore carried out in relation to this specific effect. The risk of bias assessments of non‐randomised studies considered adherence and differences in additional interventions (‘co‐interventions’) between intervention groups.

Important co‐interventions considered were interventions performed in school, during the regular school year, which are complementary to regular classes and school activities such as tutoring, short‐term reading or math interventions, or socioemotional support groups for students with a specific disability. Furthermore, we considered technological tools available to students with SEN as important co‐interventions.

##### Assessment

At least two review authors independently assessed the risk of bias for each relevant outcome from the included studies. Any disagreements were resolved by a third reviewer with content and statistical expertise and is reported in the supplementary file, which contains the risk of bias assessment for each included study outcome in the completed review.

#### 4.3.4 Measures of treatment effect

##### Continuous outcomes

All primary outcome measures in the present review were continuos. Effect sizes with 95% CIs were calculated, where means and standard deviations were available. If means and standard deviations were not available, we calculated standardised mean differences (SMDs) from *F*‐ratios, *t*‐values, *χ* ^2^ values, and correlation coefficients, where available, using the methods suggested by Wilson and Lipsey (2001). If not enough information was available, we requested this information from the principal investigators. Hedges' *g* was used for estimating SMD.

##### Dichotomous outcomes

Secondary outcome measures in the present review were dichotomous. Only one study reported secondary outcomes and they were all reported as estimated risk differences with standard errors. Not enough information was reported to calculate odds ratios as planned (Dalgaard et al., 2021) so the estimated risk differences with 95% CIs calculated using the reported standard errors were used in the data synthesis.

Excel was used for storing data.

#### 4.3.5 Unit of analysis issues

To account for possible statistical dependencies, we examined a number of issues: we assessed whether suitable cluster analysis was used, if assignment of units to treatment was clustered, whether individuals had undergone multiple interventions, whether there were multiple treatment groups or multiple time points of measurement, the mix of students, and whether several studies were based on the same data source.

##### Clustered assignment of treatment

No adjustments were necessary for clustering as the studies did not analyse whole classes (only one or a few students in a class).

##### Mixed student population

In the data synthesis, we planned to use only studies in which it was possible to extract a separate effect size for children with special needs. None of the included studies reported outcomes for a population of students consisting of both children with and without SEN.

##### Multiple intervention groups and multiple interventions per individual

In a few cases, included studies had multiple intervention groups with different individuals and one control group. However, these studies were all rated Critical risk of bias and were thus not used in the data synthesis.

##### Multiple studies using the same sample of data

Seven pairs of studies used the same sample of data, and in the meta‐analysis we only included one estimate of the effect for each outcome measure from these samples of data as outlined in the protocol. This was done to avoid dependencies between the ‘observations’ (i.e., the estimates of the effect) in the meta‐analysis. The choice of which estimate to include was based on our risk of bias assessment of the studies. We chose the estimate from the study that we judged to have the least risk of bias. Note though, that in four pairs, both studies were rated Critical risk of bias and none of the studies from these pairs could thus be used in the data synthesis, and further, in the remaining three pairs, one study each was rated critical risk of bias leaving one study from each of these three pairs to be used in the data synthesis (see section *Included studies*).

##### Multiple time points

If the results had been measured at multiple time points, each outcome at each time point was planned to be analysed in a separate meta‐analysis with other comparable studies taking measurements at a similar time point. All relevant measures in the included studies were taken at post intervention only.

#### 4.3.6 Dealing with missing data

Missing data in the individual studies was assessed using the risk of bias tool. Studies had to permit calculation of a numeric effect size and standard error for the outcomes to be eligible for inclusion in the data synthesis. Where studies had missing summary data, such as missing standard deviations, we derived these from *F*‐ratios, *t*‐values, *χ* ^2^ values, and correlation coefficients using the methods suggested by Wilson and Lipsey (2001). If not enough information was yielded to calculate an effect size and standard error, the review authors requested this information from the principal investigators. In one case it was necessary; we requested additional information from the authors, who kindly provided the necessary information.

#### 4.3.7 Assessment of heterogeneity

Heterogeneity among primary outcome studies was assessed with *χ* ^2^ (*Q*) tests, and the *I* ^2^, and *τ* ^2^ statistics (Higgins et al., 2003). Any interpretation of the Chi‐squared test was made cautiously on account of its low statistical power.

#### 4.3.8 Assessment of reporting biases

Reporting bias refers to both publication bias and selective reporting of outcome data. Here, we state how we planned to assess publication bias. We used funnel plots for information about possible publication bias.

#### 4.3.9 Data synthesis

The present review followed standard procedures for conducting systematic reviews using meta‐analysis techniques. The systematic review protocol (Dalgaard et al., 2021) was published in May 2021. The protocol is available at: https://doi.org/10.1002/cl2.1170.

The overall data synthesis was conducted where effect sizes were available or could be calculated, and where studies were similar in terms of the outcomes measured. Meta‐analysis of outcomes was conducted on each metric separately (as outlined in section ‘Types of outcome measures’).

Studies that were assessed as being in critical risk of bias were not included in the data synthesis.

As the intervention deals with diverse populations of participants (from different countries with different disabilities or SEN), and we therefore expected heterogeneity among primary study outcomes, all analyses of the overall effect were inverse variance weighted using random effects statistical models that incorporate both the sampling variance and between study variance components into the study level weights. The estimation of τ^2^ was the DerSimonian and Laird (1986) estimate (DerSimonian & Laird, 1986). Random effects weighted mean effect sizes were calculated using 95% CIs, and we provide graphical displays (forest plots) of effect sizes.

None of the studies that could be used in the meta‐analyses used the same data.

Several studies provided results separated by subscales of an outcome measurement. As there was not a sufficient number of studies included in any of the meta‐analyses to use robust variance estimation as planned; Dalgaard et al., 2021), we conducted the meta‐analyses using a synthetic effect size (the average of the subscales for a particular measurement) to avoid dependence between effect sizes.

Attempts to apply robust variance estimation were performed in STATA while all meta‐analyses were carried out in Revman 5.4.

#### 4.3.10 Subgroup analysis and investigation of heterogeneity

We aimed to investigate the following factors with the aim of explaining potential observed heterogeneity: study‐level summaries of participant characteristics (e.g., studies considering a specific disability category such as ‘learning disorders’ or ‘students with autism spectrum disorders’, or a specific gender or age group, or studies where effects are shown separately for boys and girls or by age, for example, 6–10 year old/11–16 year old/17–19 year old) if the information was available (Dalgaard et al., 2021). Furthermore, as stated in the protocol (Dalgaard et al., 2021) we aimed to explore the specific characteristics of the inclusive educational setting as outlined in the section: *The intervention* (e.g., full‐time or part‐time inclusion and involvement or not involvement of additional teaching and/or pedagogical resources).

However, due to either very limited variability between studies or too few studies reporting the moderator, this was only possible for the following moderators: a few specific disability categories (autism spectrum disorders and physical disabilities), co‐teaching (involvement of additional qualified teaching resources), and average age of participants. We attempted to conduct moderator analyses using meta‐regression applying the robust variance estimation (RVE) technique with small sample adjustment to the residuals and the Satterthwaite degrees of freedom (Satterthwaite, 1946) for tests (Tipton, 2015). However, results were untrustworthy as degrees of freedom were <4 (as suggested by Tanner‐Smith & Tipton, 2014; Tipton, 2015).

Single‐factor subgroup moderator analysis was used where possible to explore the following factors: specific disability categories (autism spectrum disorders vs. other disability categories, and physical disabilities vs. other disability categories), co‐teaching (involvement of additional qualified teaching resources vs. no involvement of additional qualified teaching resources), and average age of participants (less than 10 years of age vs. 10 years or older, and less than 15 years of age vs. 15 years or older) for outcomes where there were at least two studies in each subgroup. Thus, based on the available information within the studies which could be used in the meta‐analyses, we conducted single‐factor subgroup analyses using *child overall psychosocial adjustment* as an outcome based on the following variables: children with autism spectrum disorder versus all other types of disabilities, children with a mean age of 15 or higher versus children with a mean age less than 15, children with a mean age of 10 or higher versus children with a mean age less than 10, and finally children with physical disabilities (including severe visual impairment/blindness) versus all other types of disabilities. For the studies reporting on child overall psychosocial adjustment, there were too few studies or too limited variance in other potential moderators related to both the children and the inclusive settings to conduct any further moderator analyses. Based on the available information within the studies which could be used in the meta‐analyses, we conducted single‐factor subgroup analyses using *Math* as an outcome based on the following variables: Studies in which the inclusive setting was co‐taught by teachers with qualifications in both general and special education versus studies in which there was no co‐teaching in the inclusive setting or in which this was unclear, children with a mean age of 15 or higher versus children with a mean age less than 15, and children with a mean age of 10 or higher versus children with a mean age less than 10. For the studies reporting Math as an outcome, there were too few studies or too limited variance in other potential moderators related to both the children and the inclusive settings to conduct any further moderator analysis.

The subgroup analyses were inverse variance weighted using random effects statistical models that incorporate both the sampling variance and between study variance components into the study level weights. Random effects weighted mean effect sizes for each subgroup were calculated using 95% CIs. The assessment of any difference between subgroups was based on 95% CIs. No conclusions from single‐factor subgroup analyses were drawn and interpretation of relationships was cautious, as they were based on subdivision of studies and indirect comparisons.

#### 4.3.11 Sensitivity analysis

Sensitivity analyses were carried out by restricting a particular meta‐analysis to a subset of all studies included in the original meta‐analysis and were used to evaluate whether the pooled effect sizes were robust across components of risk of bias.

For methodological quality, we performed sensitivity analysis for the confounding, selection, classification, deviation, measurement and reporting risk of bias items of the risk of bias checklists. For the remaining items on the risk of bias checklist, there were no variations in the rating.

One study (Cosier et al., 2013) analysed inclusion as a continuous variable, that is, time spent in general education. Thus, the reported coefficients reflect the effect of a one hour increase in time spent in general education on student achievement. In the main analysis we used the effect of a standard deviation (11.6 h) increase in the time spent in general education. Sensitivity analysis was used to examine the robustness of conclusions in relation to multiplying the reported coefficient with two standard deviations increase in time spent in general education instead of one standard deviation.

##### Treatment of qualitative research

The review does not include qualitative research.

##### Summary of findings and assessment of the certainty of the evidence

See supplementary descriptive table.

## 5 RESULTS

### 5.1 Description of studies

See supplementary descriptive table.

#### 5.1.1 Results of the search

**Figure 1:**
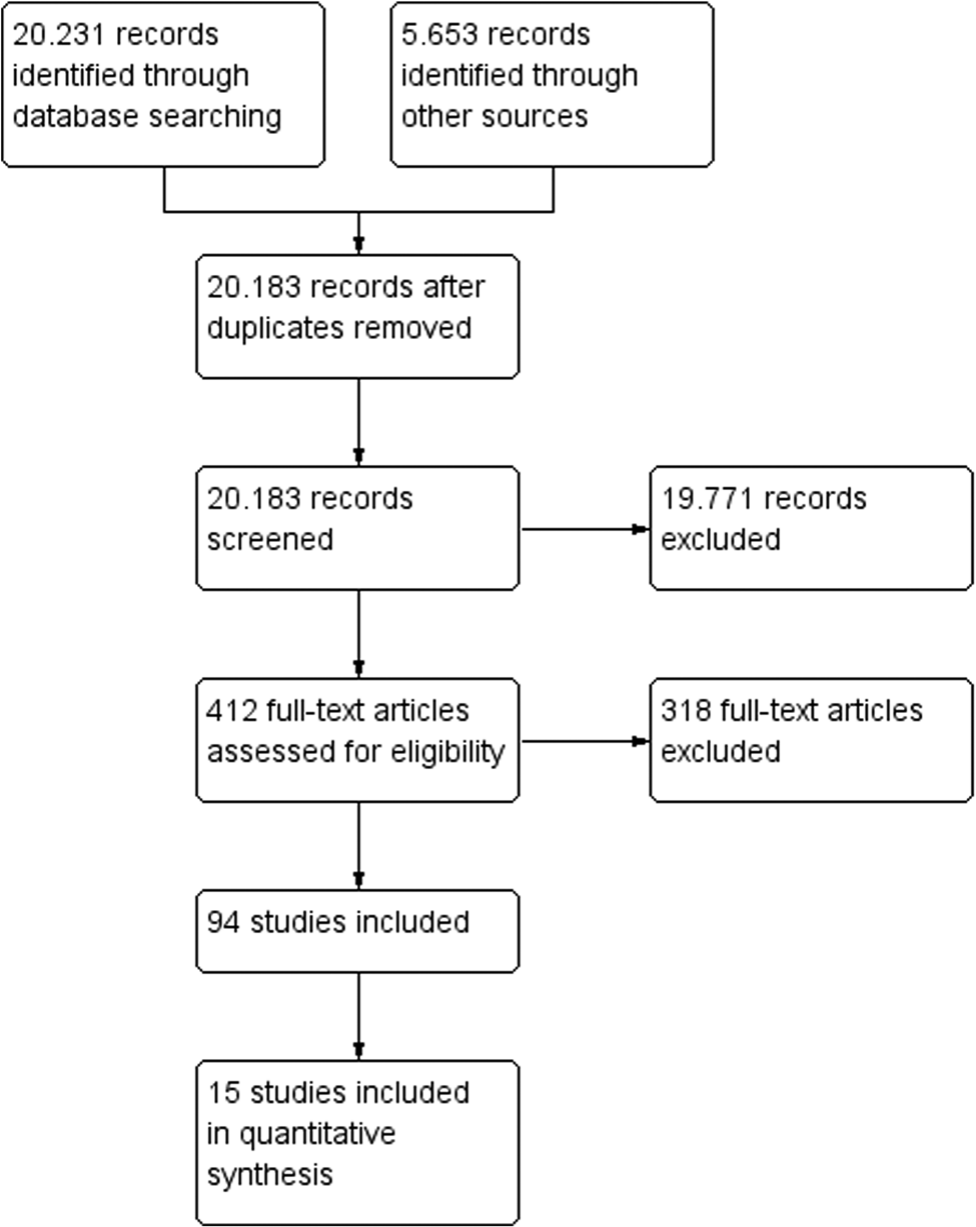
Flowchart

The results are summarised in a flow chart (Figure 1).

The total number of potential relevant records was 20.183 after excluding duplicates (database: 20.231; grey, hand search, snowballing and other resources: 5.653). All records were screened based on title and abstract; 19.771 were excluded for not fulfilling the screening criteria including 23 records that were unobtainable despite efforts to locate them through libraries and searches on the Internet. A list of these references can be found in Supporting Information: Appendix 3. Four hundred and twelve records were ordered, retrieved and screened in full text. Of these, 318 did not fulfil the screening criteria and were excluded. Ninety‐four studies were included in the review. Fifteen studies could be used in the data synthesis.

#### 5.1.2 Included studies

The search resulted in a final selection of 94 studies, which met the inclusion criteria for this review. All 94 studies were non‐randomised studies, with a comparison of two or more groups of participants, that is, at least a treated group and a control group. Descriptions of the intervention and control conditions within each included study were extracted in as much detail as possible and can be found in the supplementary descriptive table. The 94 studies analysed data from 87 different samples. Seven pairs of studies used the same sample: Buckley et al. (2006) and Buckley et al. (2007) of which one was rated overall Critical risk of bias and was therefore not used in the data synthesis; Heiman (2001) and Heiman and Olenik‐Shemesh (2015) were both rated overall Critical risk of bias and not used in the data synthesis; Jenks et al. (2009) and Jenks et al. (2012) of which one was rated overall Critical risk of bias and not used in the data synthesis; Knox (2001) and Knox and Conti‐Ramsden (2003) were both rated overall Critical risk of bias and not used in the data synthesis; Zweers et al. (2020) and Zweers et al. (2021) were both rated Critical risk of bias overall; Rainer (2014, 2015) were both rated overall Critical risk of bias and finally Reed et al. (2012) and Waddington and Reed (2017) of which one was rated Critical risk of bias and not used in the data synthesis.

Only 15 studies could be used in the data synthesis, however one study did not have any outcomes in common with the other 14 studies and could thus not be used in any of the meta‐analyses. The outcomes from this study are reported in Table 7 along with other outcomes from the other 14 studies that could not be pooled as they also were only reported in a single study. 79 studies could not be used in the data synthesis as they were judged to have Critical risk of bias (see supplementary documents for the detailed risk of bias assessments). In accordance with the protocol, we excluded studies rated overall Critical risk of bias items from the data synthesis on the basis that they would be more likely to mislead than inform.

The 94 studies came from 19 different countries, with a majority of studies from the US (49).

Of the 15 studies used in the data synthesis, four studies were from the US, three studies were from the UK, two studies were from the Netherlands, one study was from Switzerland, one study was from Finland, one study was from Germany, one study was from The Czech Republic, one study was from Belgium and one was from Denmark.

**Table 1:** Studies by country

Country	Studies included in review	Studies used in data synthesis
Australia	1	0
Belgium	1	1
Canada	1	0
Chech Republic	1	1
Denmark	2	1
Finland	1	1
Germany	6	1
Greece	1	0
Ireland	1	0
Israel	5	0
Netherlands	8	2
Norway	1	0
Poland	4	0
Slovenia	1	0
Sweden	1	0
Switzerland	1	1
Turkey	1	0
UK	8	3
USA	49	4
Total number	94	15

The main characteristics of the 15 studies used in the data synthesis are shown in Table 2.

**Table 2:** Summary of the 15 studies used in the data‐synthesis

Characteristic (number of studies reporting)		
Baseline year (9)	Average (SD)	2006 (5.02)
	Range	1998–2012
Number of children in inclusive setting (14)	Average (SD):	151 (351.14)
	Range	10–1357
Number of children in segregated placement (14)	Average (SD):	261 (719.42)
	Range	5–2752.0
Number of children total (15)	Average (SD):	471 (1056.19)
	Range	15–4109.0
Percent female, intervention group (11)	Average (SD)	37.88 (10.69)
	Range	15–52.2
Percent female, total (12)	Average (SD)	38.33 (13.92)
	Range	12–63.0
Mean age (years) children in intervention group (8)	Average (SD)	11.05 (4.04)
	Range	6.88–16.63
Mean age (years) children total (11)	Average (SD)	10.71 (3.84)
	Range	5.64–16.63

On average, the intervention baseline year was 2006, varying between 1998 and 2012. The included studies varied a lot in size, and thus the total number of children analysed in the studies ranged from 15 to 4109 children with an average of 471. Similarly, the children placed within inclusive settings within the included studies ranged from 10 to 1357 with an average of 151. The number of children in segregated settings ranged between 5 and 2752 with an average of 261. Based on the 11 studies reporting the percentage female among the children placed within inclusive settings the average was 37.88% and a little higher (38.33%) using the 12 studies reporting the percent of female students with special needs in total. Thus, the majority of children analysed are boys, which is consistent with the finding that for a number of diagnoses defined as SEN boys are more vulnerable than girls (Skårbrevik, 2002). The average age of children placed within inclusive settings was 11.05 years ranging from 6.88 to 16.63 years and a little higher (10.71) based on the 11 studies reporting the average age of students with special needs in total.

**Table 3:** Types of special needs

Study	Type of special educational needs/diagnosis
Albano, 2009	Learning disabilities (LD)
Buckley, 2006	Down Syndrome
Cole, 2004	Special needs include multiple types of disabilities or difficulties (described as mild disabilities)
Cosier, 2013	Special needs include multiple types of disabilities or difficulties
Dessemontet, 2012	Intellectual disability
Heyl, 2015	Visual impairments
Hienonen et al., 2021	Special needs include multiple types of disabilities or difficulties
Jenks, 2009	Cerebral palsy
Mrug, 2002	Physical disabilities. The most frequent diagnosis was cerebral palsy
Nash‐Aurand, 2014	Special needs include multiple types of disabilities or difficulties
Rangvid,^2021^	Special needs include multiple types of disabilities or difficulties
Reed, 2012	Autism Spectrum Disorder (ASD)
Rowley, 2012	Autism Spectrum Disorder (ASD)
Stoutjesdijk, 2016	ADHD
Tremblay, 2013	Learning disabilities (LD)

Of the 15 studies used in the data synthesis, five studies included children with multiple types of disabilities, three studies included children with learning disorders/intellectual disabilities, two studies included children with autism spectrum disorders, one study included children with ADHD, two studies included children with physical handicaps such as cerebral palsy, one study included children with visual impairments, and one study included children with Down syndrome (Table 3).

As can be seen from Table 4 the majority of included studies were of students with special needs placed in full time inclusive settings (10) while the time spend in the inclusive settings varied within five studies.

**Table 4:** Types of inclusive settings

Study	Type of inclusion
Albano, 2009	Inclusion placement where students spend 50% or more of their day with peers in the general education. No mentioning of co‐teaching.
Buckley, 2006	Full time inclusion (co‐taught as learning support assistants were present for most of the day).
Cole, 2004	Educational setting is only reported for math and reading instruction, nothing is reported regarding co‐teaching
Cosier, 2013	Average number of hours per week in general education (ranged from 0 to 40 h) no mentioning of co‐teaching.
Dessemontet, 2012	Full time inclusion. No mentioning of co‐teaching.
Heyl, 2015	Full time inclusion. No mentioning of co‐teaching.
Hienonen et al., 2021	Full time inclusion. No mentioning of co‐teaching.
Jenks, 2009	Full time inclusion. No mentioning of co‐teaching.
Mrug, 2002	Full time inclusion. No mentioning of co‐teaching.
Nash‐Aurand, 2014	Co‐taught, only reported for math classes.
Rangvid, 2021	Inclusive setting may be both full time and part time, segregated setting is defined as more than 9 h of support per week
Reed, 2012	Full time inclusion. Each class in the mainstream schools was under the supervision of a teacher with postgraduate qualifications in teaching and each class had at least one educational support staff member. Unclear if educational support staff had qualifications in special education.
Rowley, 2012	Full time inclusion. No mentioning of co‐teaching.
Stoutjesdijk, 2016	Full time inclusion. The teachers in regular schools were coached by professionals from special educational services, also including school psychologists. In addition, children in regular classrooms received support from learning support teachers (either visiting or based at the school.
Tremblay, 2013	Full time inclusion (co‐taught).

Very few the studies used in the data synthesis reported a severity measure of the disabilities of the children with special needs. However, as can be seen from Table 5, 11 out of the 15 samples used in the meta‐analyses did not include children with the most severe special needs, either because the most severe cases were removed from the analyses (six studies) or because eligibility criteria for the study excluded children with more severe disabilities (five studies). This finding should be taken into consideration when interpreting findings from the meta‐analysis.

**Table 5:** Severity selection/exclusion

Study	Disability categori	Information on selection and/or exclusion criteria	Removed the most severe cases	The sample not severe (by eligibility criteria)	No information	Do not select or exclude on severity
Albano, 2009	Learning disability	Inclusion criteria include: (1) having a discrepancy of 18 points or greater between their IQ and their achievement, average IQ (85–115); and (3) having consistent school failure. All should be working towards a high school diploma.		1		
Buckley, 2006	Down Syndrome	The five ‘least able’ teenagers from the special schools were taken out of the comparison group, before the two groups were compared. These five ‘least able’ teenagers are those with significantly more developmental delay and health problems than the rest of the group. Further reported on p. 55: ‘Two of them have autism in addition to Down syndrome and 3 of the 5 have significantly high rates of difficult behaviours. These young people have had multiple difficulties since childhood, and children with this level of difficulty would not have been placed in mainstream classes in any part of the county at the time of the study'.	1			
Cole, 2004	Mostly LD (70%) and mild mental disability (20%) (and 6‐7% with emotinal disability)	Only students identified with mild disabilities are eligible		1		
Cosier, 2013	Multiple	All participants in the analysis for this article were age 6 or older during the 2007 academic school year and labelled as having one of the federal categories of disability covered under Individuals With Disabilities Education Act (IDEA), or additional categories including (a) suspected of having a disability, (b) being at risk for a disability, (c) ‘other’, and (d) ‘child does not have an Individualized Education Program (IEP)’. The latter category indicates either the child was (a) declassified as having a disability or (b) was determined ineligible to receive special education services and was being served via Section 504 of the Rehabilitation Act (1973).			1	
Dessemontet, 2012	Intellectual disability	All the included (treated) children with high levels of functioning were excluded and all the children in special schools (control) with low levels of functioning were excluded (level for exclusion not reported and ‘functioning’ is probably IQ, academic achievement [pre test scores] as well as adaptive skills [pre test scores]).	1			
Heyl, 2015	Visual impairments	From low impairment to blind and Additional disabilities were present in nearly half of the children (more than half of the group had slight to severe intellectual disabilities; not shown in Table 1). There were more children with additional disabilities at special schools.				1
Hienonen et al., 2021	Not reported	They match and cognitive skills are part of the matching criteria and they loose 68% (special class is their treatment and thus are the ones matched). All are Tier 3 and Tier 3 students study school subjects either according to the general curriculum (55%) or to an individualised syllabus in one or more subjects (45%), depending on the severity and nature of the disability (OSF, 2019). Curriculum was used as a covariate. Special education teachers were asked to complete a questionnaire about whether a student studied according to a general or individualised curriculum. For this, we created a categorical ordered variable for curriculum according to national statistics (0 = general curriculum). Number of students with individualised curriculum descreases due to matching	1			
Jenks, 2009	Cerebral palsy	All parents with children with cerebral palsy (a formal diagnosis of cerebral palsy and a verbal intelligence quotient (IQ) of at least 70) about to enter first grade were invited.		1		
Mrug, 2002	Physical disabilities. The most frequent diagnosis was cerebral palsy	Selection criteria were: 11 years of age or older, attending a primary or secondary school, having a visible physical disability present before 4 years of age, and being able to comprehend the questionnaire.				1
Nash‐Aurand, 2014	Multiple	Students included in this study had the following IDEIA defined eligibilities: specific learning disabilities (SLD), mild intellectual disabilities (MID), vision impairments (VI), deafness (D), hearing impairments (HI), emotional/behavioural disorders (EBD), autism (ASD), and other health impairments (OHI). Most were SLD, OIH and ASD. Selection into the study was based on availability of test results (ordinary tests, i.e., probably no individualised curriculum/education plans)		1		
Rangvid, 2021	Multiple	The treated (students mainstreamed) are matched to the control group (students not mainstreamed), less than 1% of treated are lost to matching and slightly less than 60% of the control group is retained.	1			
Reed, 2012	Autism	Participants were recruited from across the UK, however within the initial sample there were large imbalances (eg the autism symptom severity was higher for students attending special schools as would be expected) and so they used a median split procedure to exclude students at the more severe end of the spectrum to increase the balance on confounders between the two groups.	1			
Rowley, 2012	Autism	The subsample (100) were drawn from a big cohort, with no explanation on how they drew them, other than it was the first 100 (from a total of 117) for which an ADOS Module 3 s was available. In a small number of cases (*N* = 11), it was not possible to assign friendship or bullying scores, since the child's verbal account was too limited or ambiguous to allow for accurate scoring and they were excluded from the analysis.	1			
Stoutjesdijk, 2016	ADHD	Selection criteria are clearly defined: children have to be eligible for special educational support by meeting the official admission criteria, children must score in the clinical range on the ADHD Total subscale of the Social Emotional Questionnaire, and children must have a full‐scale IQ of 85 or higher (within or above normal range, WISC‐R). Do not report the numbers in each condition in the main sample. From the main sample of 180 children, 64 children were selected		1		
Tremblay, 2013	Learning disabilities (LD)	Level of impairment not considered other than all are LD students defined as: despite having normal levels of intelligence, hearing, and sight, they present difficulties in language or speech development and/or the acquisition of reading, writing, or calculation, with a level of gravity requiring specific intervention which regular instruction alone cannot provide			1	
Total			6	5	2	2

#### 5.1.3 Excluded studies

42 studies were initially included but were excluded later with reasons. A list of the late‐stage excluded studies can be found in the references.

### 5.2 Risk of bias in included studies

The detailed risk of bias coding for each of the 94 studies is available in a supplementary document.

Summary scores from the risk of bias assessment are shown in Table 6. All 94 studies were non‐randomised studies and were rated using the ROBINS‐I tool. If a study reported on several outcomes, a risk of bias assessment was carried out for each of those outcomes. If ratings differed, only the most favourable assessment is reported in Table 6, since this assessment may have been a reason to include the study in the meta‐analysis. As stated in the protocol, we stopped the assessment of a non‐randomised study outcome when it was rated ‘Critical’ on any of the items, therefore not all studies are rated on all domains.

**Table 6:** Summary risk of bias assessment

Judgement: Risk of Bias Item:	Low	Moderate	Serious	Critical	No information	Number of studies rated on this domain
Overall Judgement		1	14	79		94
Confounding Bias		3	20	69		92
Selection Bias	1	9	13	1		24
Classification Bias	8	5	3	3		19
Deviation Bias	6	2	1		7	16
Missing Data	12	2			2	16
Measurement bias	5	9	2			16
Reporting Bias		14	2			16

Seventy‐nine studies were rated with an overall critical risk of bias, corresponding to a risk of bias so high that the findings should not be considered in the data synthesis. The overall Critical risk of bias rating was mainly due to issues on the Confounding bias item; 69 were rated Critical risk of bias on this item; that is, they failed to establish a comparison group that was balanced on important confounders and further either did not control for any confounders. One study had a critical risk of selection bias, three studies had a critical risk of classification bias (of which two also were rated Critical on confounding) and seven studies were judged to be in critical risk of bias due to ratings of serious risk of bias in multiple domains (six on confounding and selection bias and one on confounding, selection, measurement and reporting bias).

Fourteen studies were rated with an overall serious risk of bias, and one study with an overall moderate risk of bias.

Of the 25 studies not rated Critical risk on the Confounding item, 20 had Serious issues on this item, three were rated Moderate risk of bias and two were not rated on this item. Besides the one study rated Critical risk of bias on the Selection bias item, one study was rated low risk of bias, nine were rated Moderate risk of bias and 13 were rated Serious risk of bias. Besides the three studies rated Critical risk of bias on the Classification bias item, eight studies were rated Low risk of bias, five were rated Moderate risk of bias and three were rated Serious risk of bias. Six studies were rated Low risk of bias on the Deviation item, two were rated Moderate and one was rated Serious risk of bias. On the Missing data item 12 were rated Low risk of bias and one was rated Moderate risk of bias. On the Measurement item, five were rated Low risk of bias, nine were rated Moderate and two were rated Serious risk of bias. Fourteen studies were rated Moderate risk of bias on the Selection of Reported Results mainly because there was no a priori analysis plan and a further two were rated Serious risk of bias as they had other issues in addition to no a priori analysis plan.

The risk of bias assessment thus left us with only 15 studies which could be used in the data‐synthesis, 14 with an overall Serious risk of bias rating and one study which was rated as in overall Moderate risk of bias.

### 5.3 Effects of interventions

Fifteen studies provided standardised mean differences and variances or data that enabled calculation of standardised mean differences and variance effect estimates that could be used in the data synthesis. No adjustments were necessary for clustering as the studies did not analyse whole classes (only one or a few students in a class).

One study (Cosier, 2013) analysed inclusion as a continuous variable, that is, time spent in general education. Thus, the reported coefficients reflect the effect of a 1‐h increase in time spent in general education on student achievement. Mean time spent in general education as well as the standard deviation of the time spent in general education was reported. We will use the effect of a standard deviation increase in the time spent in general education in the data synthesis and investigate the robustness of results in the sensitivity analysis. Thus, the results of the study Cosier (2013) will reflect an increase in 11.6 h spent in general education.

### 5.4 Meta‐analysis

#### 5.4.1 Child outcomes

##### Overall psychosocial adjustment

**Figure 2:**
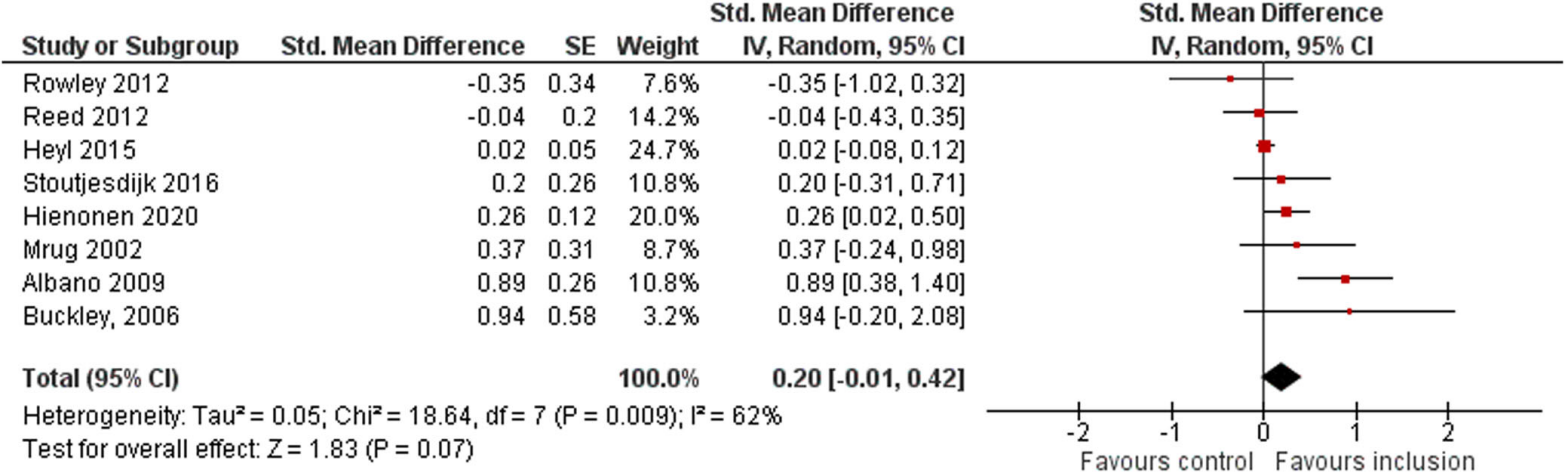
Meta‐analyses: Overall psychosocial adjustment

Eight studies analysed the effect of inclusion on the overall psychosocial adjustment of children with special needs as reported by themselves, by their parents or by teachers. Measures used include: Vineland Adaptive Behaviour Scale, Socialisation Subscale; Teacher Report Form; Self‐Perception Profile for Adolescents; The Personality Assessment Questionnaire; The Strengths and Difficulties Questionnaire; ADOS‐G, module 3: Child self‐report; Learning motivation based on the Finnish LTL framework subscale for Avoidance Orientation. The random effects weighted standardised mean difference favouring the intervention group was 0.20 (95% CI: −0.01 to 0.42) and not statistically significant. The forest plot is displayed in Figure 2. There is some heterogeneity between the studies; the estimated *τ* ^2^ is 0.05, *Q* = 18.64, *df* = 7 and *I* ^2^ is 62% as displayed in Figure 2. Although the heterogeneity as indicated by *I* ^2^ was moderate, the estimate of *τ* ^2^ indicates that the evidence for this outcome is inconsistent, in the sense that the distribution of effect sizes spans both positive and negative effect sizes. To ease interpretation concerning the implication of heterogeneity, we calculated a 95% prediction interval. A prediction interval reflects the variation in treatment effects over different settings, including what effect is to be expected in future primary studies in similar settings. The range was (95% PI: −0.41 to 0.81) and based on this prediction interval we calculated the probability that inclusion in a future setting will have a true‐negative effect to be 22%.

##### Language and literacy

**Figure 3:**
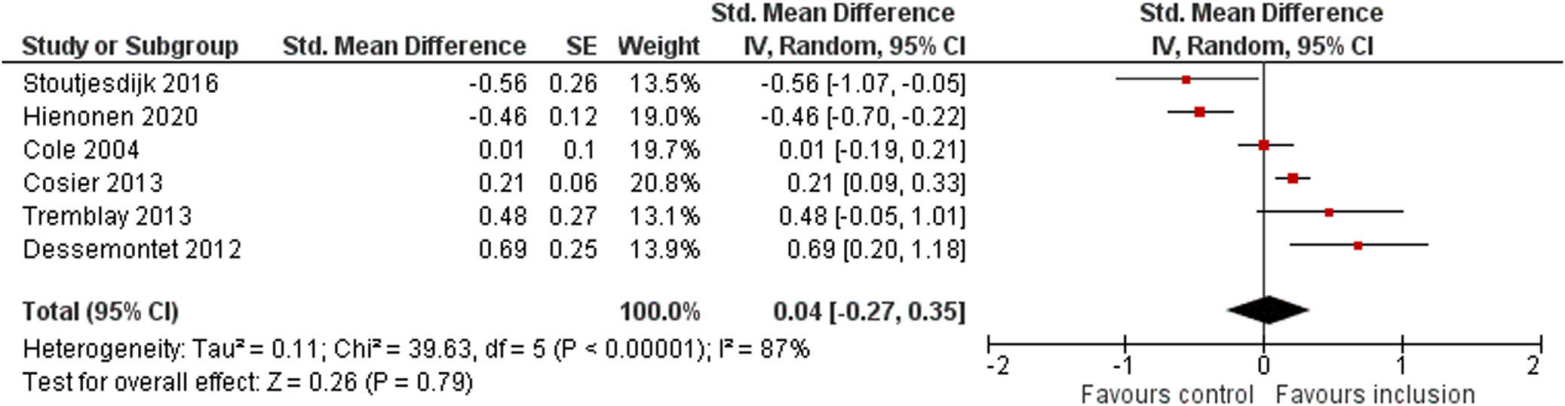
Meta‐analyses: Language and Literacy

Six studies analysed the effect of inclusion on language and literacy learning outcomes as measured by standardised and curriculum based tests. The random effects weighted standardised mean difference was 0.04 favouring the intervention (95% CI: −0.27 to 0.35) and not statistically significant. The forest plot is displayed in Figure 3. There is some heterogeneity between the studies; the estimated *τ* ^2^ is 0.11, *Q* = 39.63, *df* = 5 and *I* ^2^ is 87% as displayed in Figure 3. Although the average effect of inclusion was close to a null value it reflects that there were both large negative and large positive effect sizes in the primary studies. The prediction interval (95% PI: −0.98 to 1.06) also reflects that the study effects were dispersed over a wide range. The probability that inclusion in a future setting will have a true‐negative effect was estimated to be 46%.

##### Math

**Figure 4:**
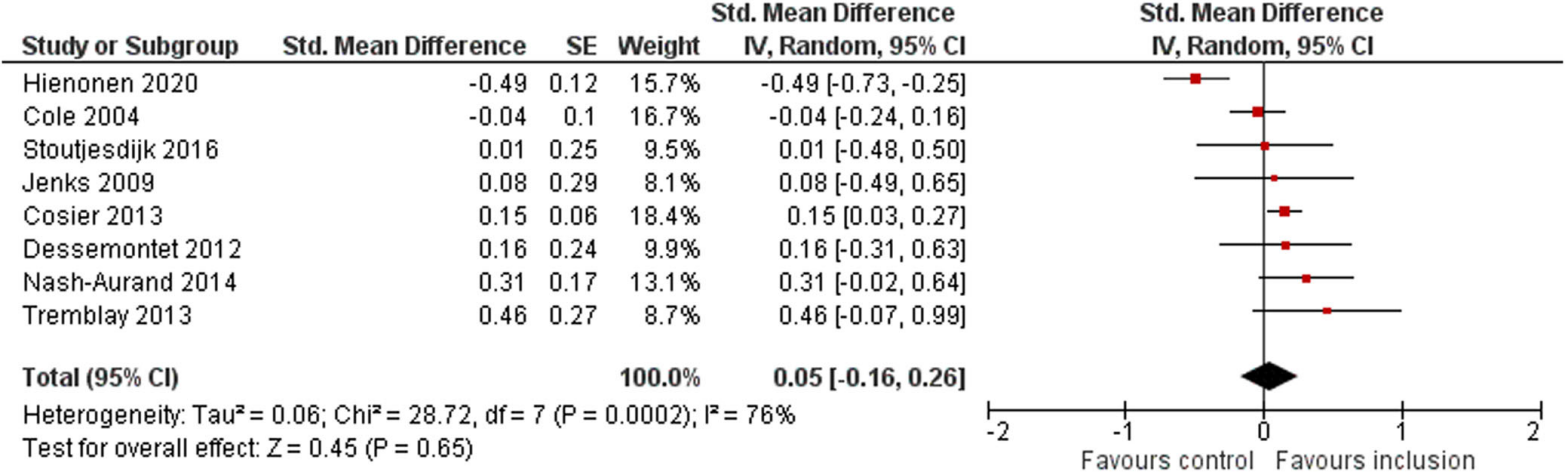
Meta‐analyses: Math

Eight studies analysed the effect of inclusion on math learning outcomes as measured by standardised and curriculum‐based tests. The random effects weighted standardised mean difference was 0.05 favouring the intervention (95% CI: −0.16 to 0.26) and not statistically significant. The forest plot is displayed in Figure 4. There is some heterogeneity between the studies; the estimated *τ* ^2^ is 0.06, *Q* = 28.72, *df* = 7 and I^2^ is 76% as displayed in Figure 4. The average effect of inclusion was close to zero, but the primary study effects were dispersed over a wide range, giving a prediction interval of (95% PI: −0.60 to 0.70) and the probability that inclusion in a future setting will have a true‐negative effect was estimated to be 43%.

##### Other outcomes

A number of relevant outcomes which we could not synthesise in the meta‐analyses, as they were only reported in a single study each, were extracted. The outcomes were measures on academic tests other than language/literacy and math, learning motivation, parent and teacher reported SDQ on friendship and bullying and socialisation skills. The effect sizes and 95% CIs are reported in Table 7.

**Table 7:** Additional outcomes

Study	Measure	Outcome	SMD [95% CI]
Hienonen et al., 2021	Average of Finnish, mathematics, foreign language, and science	GPA	−0.42 [−0.66, −0.18]
Hienonen et al., 2021	Ninth grade test	Foreign language	−0.26 [−0.50, −0.02]
Hienonen et al., 2021	Ninth grade test	Science	−0.33 [−0.57, −0.09]
Hienonen et al., 2021	Learning motivation scales	Mastery extrensic orientation	−0.31 [−0.55, −0.07]
Hienonen et al., 2021	Learning motivation scales	Perfomance approach orientation	−0.28 [−0.52, −0.04]
Hienonen et al., 2021	Learning motivation scales	Mastery intrinsic orientation	0.22 [−0.02, 0.46]
Hienonen et al., 2021	Learning motivation scales	Performance avoidance orientation	0.07 [−0.17, 0.31]
Rowley, 2012	SDQ‐P	Friendships	0.02 [−0.61, 0.65]
Rowley, 2012	SDQ‐P	Fighting/bullying	0.84 [0.21, 1.47]
Rowley, 2012	SDQ‐P	Victimisation (being bullied)	0.05 [−0.62, 0.72]
Rowley, 2012	SDQ‐T	Friendships	0.04 [−0.65, 0.73]
Rowley, 2012	SDQ‐T	Fighting/bullying	0.31 [−0.42, 1.04]
Rowley, 2012	SDQ‐T	Victimisation (being bullied)	−0.76 [−1.41, −0.11]
Rowley, 2012	ADOS‐G, module 3: Child self‐report	Friendships	−0.69 [−2.34, 0.96]
Reed, 2012	Vineland Adaptive Behaviour Scale (VABS)	Socialisation skills	0.85 [0.44, 1.26]

			Risk difference [95% CI]
Rangvid, 2021	.	Enrolment in a qualifying upper secondary education programme year 1 after secondary education	0.21 [0.16, 0.25]
Rangvid, 2021	.	Enrolment in a qualifying upper secondary education programme year 2 after secondary education	0.15 [0.11, 0.20]
Rangvid, 2021	.	Enrolment in a qualifying upper secondary education programme year 3 after secondary education	0.14 [0.07, 0.20]
Rangvid, 2021	.	Take school leaving exam	0.32 [0.30, 0.34]
Rangvid, 2021	.	Pass school leaving exam	0.29 [0.26, 0.32]
Rangvid, 2021	.	Pass school leaving exam and specific requirement for vocational education	0.24 [0.21, 0.27]
Rangvid, 2021	.	Pass ‘minimum competency threshold’ in core subjects (grade 4)	0.09 [0.05, 0.12]

Abbreviations: CI, confidence interval; SMD, standardised mean difference.

##### Secondary outcomes

One study (Rangvid, 2021) reported school completion rates (take school‐leaving exam and pass school‐leaving exam). The effect sizes and 95% CIs are reported in Table 7.

#### 5.4.2 Follow‐up outcomes

Unfortunately, none of the included studies reported follow‐up outcomes on relevant measures.

#### 5.4.3 Single‐factor sub‐group analysis

The included studies differed in terms of their child characteristics and types of inclusive settings. With between six and eight studies in a single meta‐analysis, the statistical power to detect heterogeneity of effects was not high; nevertheless, evidence of statistical heterogeneity was found in all meta‐analyses. Due to missing data and invariability between studies on most of the moderators we planned to investigate, it was only possible to investigate the impact of the following moderators: type of disability (only for the outcome child overall psychosocial adjustment); mean age (only for the outcomes: child overall psychosocial adjustment and math) and finally co‐teaching (only for the outcome math). We only performed subgroup analysis if there were at least two studies in each subgroup, for a particular outcome. All the results rely strictly on variation between studies, not within. Making inferences about different effect sizes among subgroups on the basis of between‐study differences entails a higher risk compared to inferences made on the basis of within‐study differences (Oxman, 1992). One should therefore be careful when interpreting estimates that rely on variation between studies. We have drawn no overall conclusion because the analysis is based on a subdivision of studies and indirect comparisons. The assessment of any difference between the subgroups is based on 95% CIs (if there is overlap of CIs or not) and interpretation of relationships is cautious and not based on statistical significance of subgroup average effect sizes.

#### 5.4.4 Child overall psychosocial adjustment

##### Co‐teaching

Due to invariability between studies on this moderator it was not possible to conduct a subgroup analysis.

##### Autism spectrum disorder/other types of special needs

**Figure 5:**
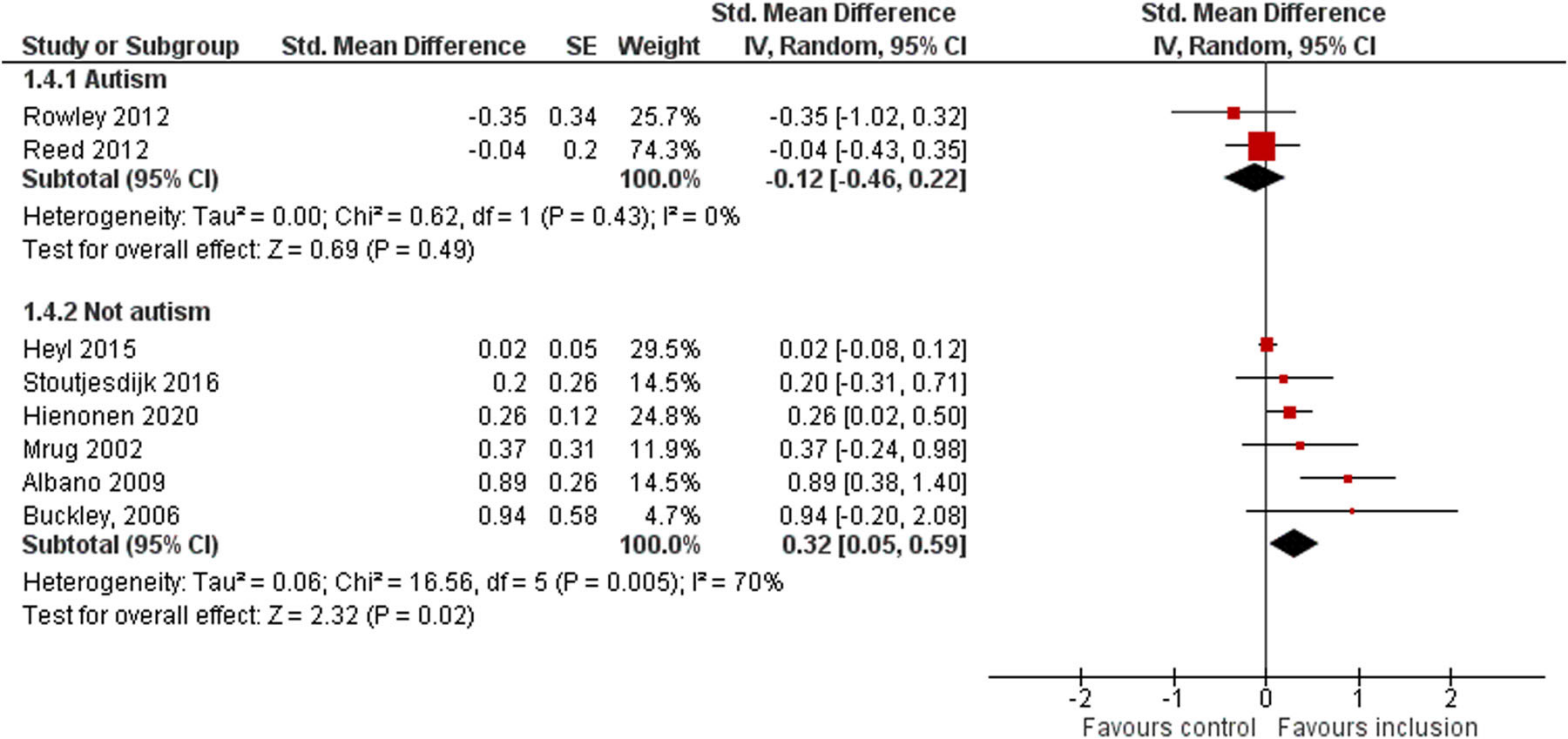
Subgroup analyses, autism spectrum disorder/other types of special needs

Of the eight studies providing effect estimates of overall child psychosocial adjustment, two studies used a sample of children with autism spectrum disorder only and six used samples of children with other types of special needs. The forest plot for the eight effect estimates is displayed in Figure 5. Pooled results for the two subgroups showed a statistically non‐significant negative effect of −0.12 (95% CI: −0.46 to 0.22) for children with autism spectrum disorder and a statistically significant positive effect of 0.32 (95% CI: 0.05 to 0.59) for children with other types of disabilities. There was no heterogeneity of effects among studies in the subgroup of children with autism spectrum disorder (*τ* ^1^ = 0.00, *I* ^2^ = 0%) while there was some heterogeneity for children with other types of special needs (*τ* ^2^ = 0.06, *I* ^2^ = 70%). The CIs of the subgroups overlapped. There was no evidence to support the hypothesis that the effect differs for children with or without autism spectrum disorders.

##### Children with physical disabilities (including severe visual impairment/blindness)/all other types of disabilities

**Figure 6:**
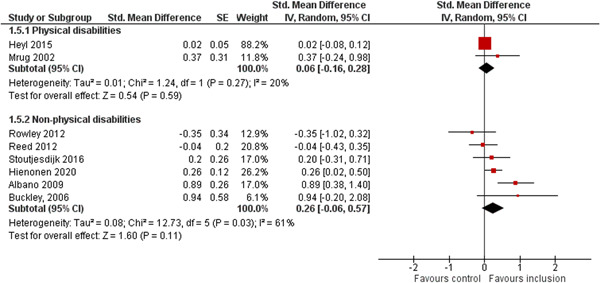
Subgroup analyses, children with physical disabilities (including severe visual impairment/blindness)/all other types of disabilities

Of the eight studies providing effect estimates of the overall child psychosocial adjustment, two studies used a sample of children with physical disabilities and six used samples of children with other types of special needs. The forest plot for the eight effect estimates is displayed in Figure 6. Pooled results for the two subgroups showed a statistically non‐significant negative effect of −0.06 (95% CI: −0.16 to 0.28) for children with physical disabilities and a statistically non‐significant positive effect of 0.26 (95% CI: −0.06 to 0.57) for children with other types of disabilities. There was limited heterogeneity of effects among studies in the subgroup of children with physical disabilities (*τ* ^2^ = 0.01, *I* ^2^ = 20%) while there was some heterogeneity for children with other types of special needs (*τ* ^2^ = 0.08, *I* ^2^ = 61%). The CIs of the subgroups overlapped. There was no evidence to support the hypothesis that the effect differs for children with or without physical disabilities.

##### Child mean age 15 or higher/child mean age less than 15

**Figure 7:**
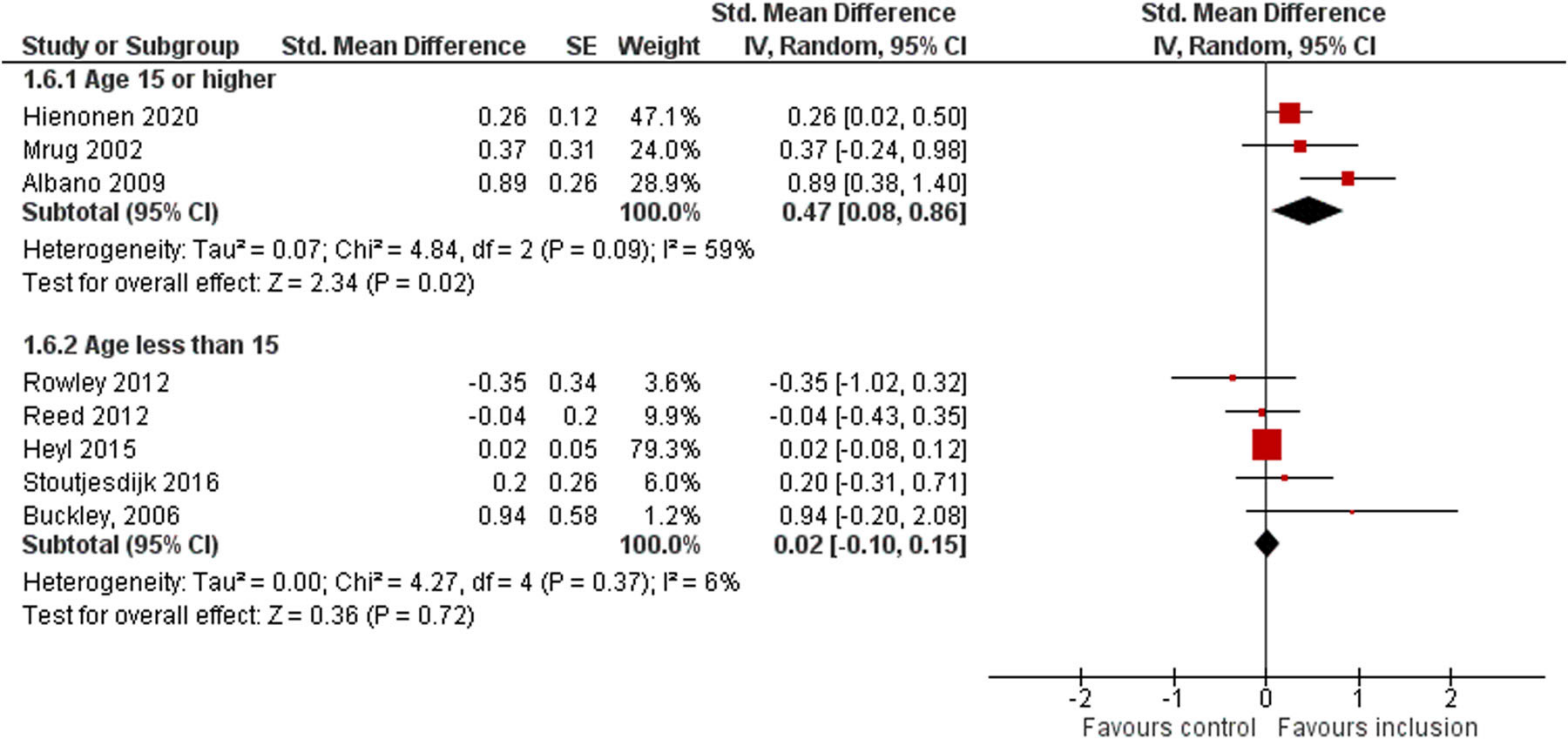
Subgroup analyses, child mean age 15 or higher/child mean age less than 15

Of the eight studies providing effect estimates of the overall child psychosocial adjustment, three studies used a sample of children with a mean age of 15 or higher and five used samples of children with a mean age less than 15. The forest plot for the eight effect estimates is displayed in Figure 7. Pooled results for the two subgroups showed a statistically significant positive effect of 0.47 (95% CI: 0.08 to 0.86) for children aged 15 or higher and a statistically non‐significant positive effect of 0.02 (95% CI: −0.10 to 0.15) for children younger than 15. There was some heterogeneity of effects among studies in the subgroup of children aged 15 or older (*τ* ^2^ = 0.07, *I* ^2^ = 59%) while there was very little heterogeneity for children younger than 15 (*τ* ^2^ = 0.00, *I* ^2^ = 6%). The CIs of the subgroups overlapped. There was no evidence to support the hypothesis that the effect differs for children aged 15 or higher versus children younger than 15.

##### Child mean age 10 or higher/child mean age less than 10

**Figure 8:**
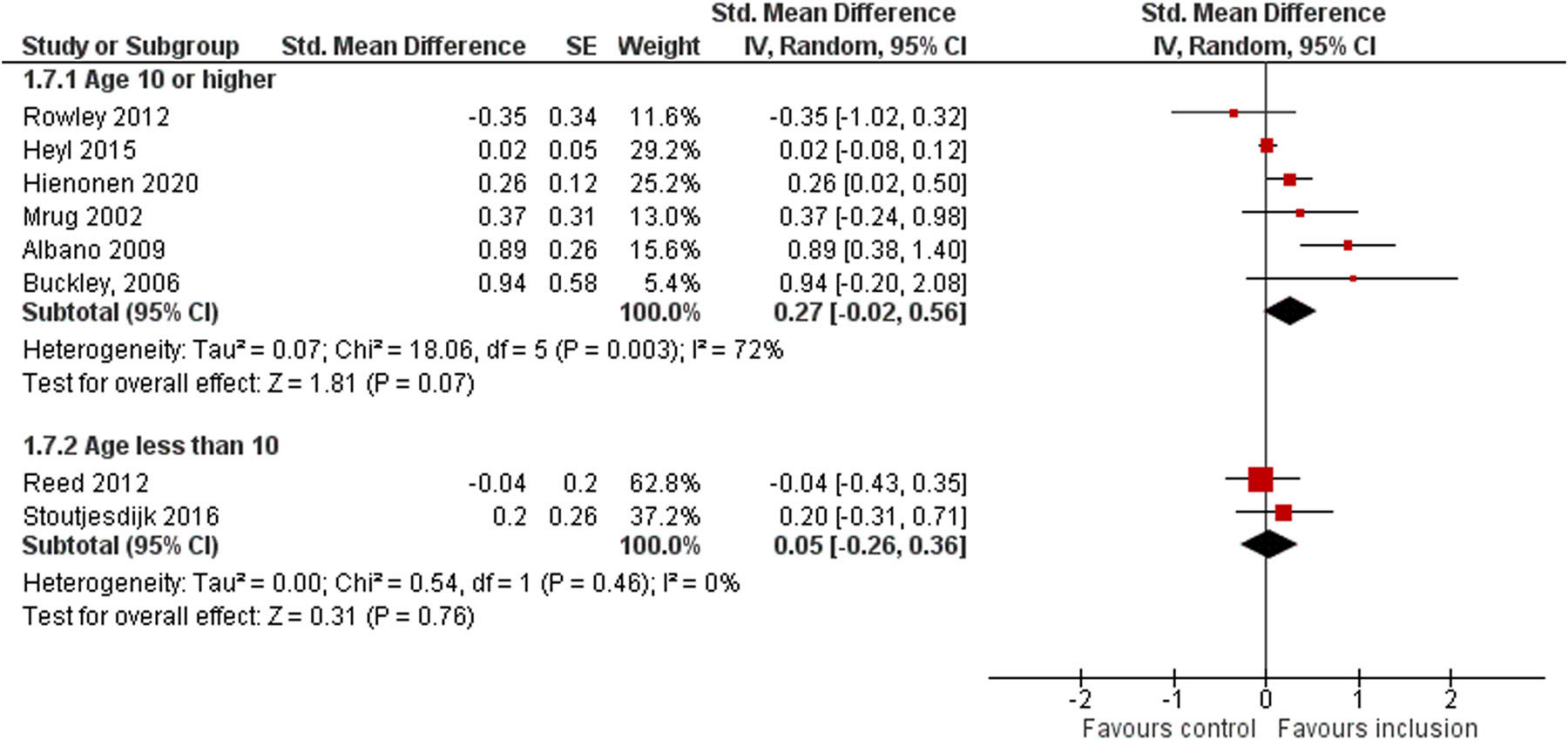
Subgroup analyses, child mean age 10 or higher/child mean age less than 10

Of the eight studies providing effect estimates of the overall child psychosocial adjustment, six studies used a sample of children with a mean age of 10 or higher and two studies used samples of children with a mean age less than 10. The forest plot for the eight effect estimates is displayed in Figure 8. Pooled results for the two subgroups showed a statistically non‐significant positive effect of 0.27 (95% CI: −0.02 to 0.56) for children aged 10 or higher and a statistically non‐significant positive effect of 0.05 (95% CI: −0.26 to 0.36) for children younger than 10. There was some heterogeneity of effects among studies in the subgroup of children aged 10 or older (*τ* ^2^ = 0.07, *I* ^2^ = 72%) while there was no heterogeneity for children younger than 15 (*τ* ^2^ = 0.00, *I* ^2^ = 0%). The CIs of the subgroups overlapped. There was no evidence to support the hypothesis that the effect differs for children aged 10 or higher versus children younger than 10.

#### 5.4.5 Language and literacy

Due to invariability between studies on the moderators it was not possible to conduct subgroup analysis using language and literacy outcomes.

#### 5.4.6 Math

##### Co‐teaching

**Figure 9:**
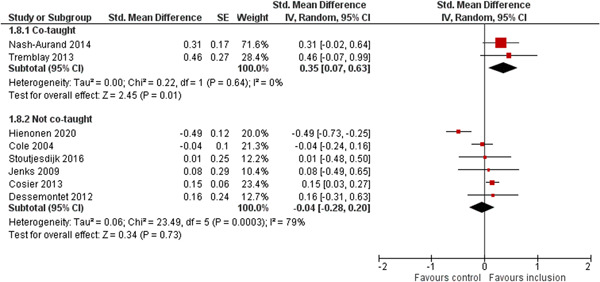
Subgroup analyses, co‐teaching

Of the eight studies providing effect estimates of math outcomes, two studies reported on an inclusive setting which was co‐taught by teachers with qualifications in both general and special education and six studies had no mentioning of co‐teaching in the inclusive setting or were unclear as to whether co‐teaching occurred. It should be noted that it is possible that in some of the former studies, there was some form of co‐teaching in the inclusive setting, which was just not specified in the published studies. The forest plot for the eight effect estimates is displayed in Figure 9. Pooled results for the two subgroups showed a statistically significant positive effect of 0.35 (95% CI: 0.07 to 0.63) for children in inclusive settings with co‐teaching and a statistically non‐significant negative effect of −0.04 (95% CI: −0.28 to 0.20) for children in inclusive settings in which there was no description of co‐teaching. There was no heterogeneity of effects among studies in the subgroup of children in inclusive settings with co‐teaching (*τ* ^2^ = 0.00, *I* ^2^ = 0%) while there was some heterogeneity for children in inclusive settings in which there was no description of co‐teaching (*τ* ^2^ = 0.06, *I* ^2^ = 79%). The CIs of the subgroups overlapped. There was no evidence to support the hypothesis that the effect differs based on whether there is co‐teaching in the inclusive setting.

##### Autism spectrum disorder/other types of special needs

Due to invariability between studies on this moderator it was not possible to conduct a subgroup analysis.

##### Children with physical disabilities (including severe visual impairment/blindness)/all other types of disabilities

Due to invariability between studies on this moderator it was not possible to conduct a subgroup analysis.

##### Child mean age 15 or higher/child mean age less than 15

**Figure 10:**
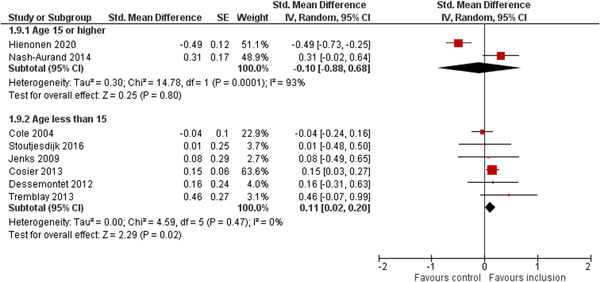
Subgroup analyses, child mean age 15 or higher/child mean age less than 15

Of the eight studies providing effect estimates of math outcomes, two studies used a sample of children with a mean age of 15 or higher and six used samples of children with a mean age less than 15. The forest plot for the eight effect estimates is displayed in Figure 10. Pooled results for the two subgroups showed a statistically non‐significant negative effect of −0.10 (95% CI: −0.88 to 0.68) for children aged 15 or higher and a statistically significant positive effect of 0.11 (95% CI: 0.02 to 0.20) for children younger than 15. There was a substantial heterogeneity of effects among studies in the subgroup of children aged 15 or older (*τ* ^2^ = 0.30, *I* ^2^ = 93%) while there was no heterogeneity for children younger than 15 (*τ* ^2^ = 0.00, *I* ^2^ = 0%). The CIs of the subgroups overlapped. There was no evidence to support the hypothesis that the effect differs for children aged 15 or higher versus children younger than 15.

##### Child mean age 10 or higher/child mean age less than 10

Of the eight studies providing effect estimates of math outcomes, two studies used a sample of children with a mean age of 10 or higher and six used samples of children with a mean age less than 10. The studies with children with a mean age of 10 and older are the same as the studies with a mean age of 15 and older. The subgroup effects are thus identical to the subgroups analysed above. There was no evidence to support the hypothesis that the effect differs for children aged 10 or higher versus children younger than 10.

#### 5.4.7 Sensitivity analysis

Sensitivity analyses were planned to evaluate whether the pooled effect sizes were robust across study design and components of methodological quality.

No randomised controlled trials were included in the meta‐analyses, so the impact of study design could not be evaluated. For methodological quality, we carried out sensitivity analyses for the confounding, selection, classification, measurement and reporting risk of bias items of the risk of bias checklists. We examined the robustness of our conclusions when we excluded studies with a serious risk of bias assessment.

The analyses are performed separately by outcome, essentially replicating the meta‐analyses conducted in Analysis 1.1, Analysis 1.2 and Analysis 1.3. We further examined the robustness of our conclusions when we increased the reported effects from a one standard deviation increase in time spent in general education to an increase of two standard deviations increase in time spent in general education in the study using time spent in general education as a continuous variable.

**Figure 11:**
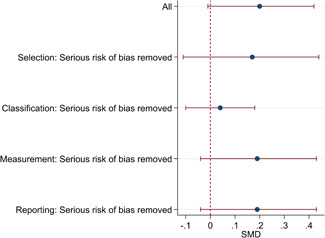
Sensitivity analysis

**Figure 12:**
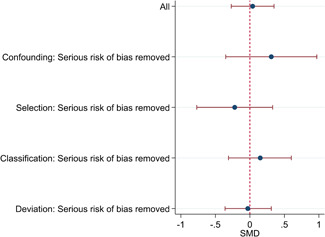
Sensitivity analysis

**Figure 13:**
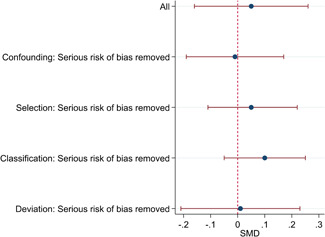
Sensitivity analysis

**Figure 14:**
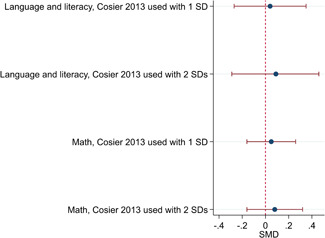
Sensitivity analysis

The results of the sensitivity analyses are provided in Tables 8, 9, 10, 11 and displayed in Figures 11, 12, 13, 14.

**Table 8:** Sensitivity analysis: Child overall psychosocial

	Number of studies	Mean	95% CI	
Studies excluded	*k*	SMD	Lower	Upper
All	8	0.20	−0.01	0.42
Overall: All studies were rated serious	‐	‐	‐	
Confounding: All studies were rated serious	‐	‐	‐	
Selection: Serious risk of bias removed	6	0.17	−0.11	0.44
Classification: Serious risk of bias removed	6	0.04	−0.10	0.18
Deviation: No study was rated serious		‐	‐	‐
Missing data: No study was rated serious		‐	‐	‐
Measurement: Serious risk of bias removed	7	0.19	−0.04	0.43
Reporting: Serious risk of bias removed	7	0.19	−0.04	0.43

Abbreviations: CI, confidence interval; SMD, standardised mean difference.

**Table 9:** Sensitivity analysis: Language and literacy

	Number of studies	Mean	95% CI	
Studies excluded	*k*	SMD	Lower	Upper
All	6	0.04	−0.27	0.35
Overall: all except one study were rated serious	‐	‐	‐	‐
Confounding: Serious risk of bias removed	2	0.31	−0.35	0.97
Selection: Serious risk of bias removed	2	−0.22	−0.77	0.33
Classification: Serious risk of bias removed	4	0.15	−0.31	0.60
Deviation: Serious risk of bias removed	5	−0.03	−0.36	0.31
Missing data: No study was rated serious	‐	‐	‐	‐
Measurement: No study was rated serious	‐	‐	‐	‐
Reporting: No study was rated serious	‐	‐	‐	‐

Abbreviations: CI, confidence interval; SMD, standardised mean difference.

**Table 10:** Sensitivity analysis: Math

	Number of studies	Mean	95% CI	
Studies excluded	*k*	SMD	Lower	Upper
All	8	0.05	−0.16	0.26
Overall: all except one study were rated serious	‐	‐	‐	‐
Confounding: Serious risk of bias removed	2	−0.01	−0.19	0.17
Selection: Serious risk of bias removed	4	0.05	−0.11	0.22
Classification: Serious risk of bias removed	6	0.10	−0.05	0.25
Deviation: Serious risk of bias removed	7	0.01	−0.21	0.23
Missing data: No study was rated serious	‐	‐	‐	‐
Measurement: No study was rated serious	‐	‐	‐	‐
Reporting: No study was rated serious	‐	‐	‐	‐

Abbreviations: CI, confidence interval; SMD, standardised mean difference.

**Table 11:** Sensitivity analysis time spend in general education

	SMD	Lower	Upper
*Sensitivity: Language and literacy*			
Cosier, 2013 used with 1 SD change in general education	0.04	−0.27	0.35
Cosier, 2013 used with 2 SDs change in general education	0.09	−0.29	0.46
*Sensitivity: Math*			
Cosier, 2013 used with 1 SD change in general education	0.05	−0.16	0.26
Cosier, 2013 used with 2 SDs change in general education	0.08	−0.16	0.32

Abbreviation: CI, confidence interval.

There were no appreciable changes in the results following removal of any of the studies nor were there any appreciable changes of doubling the effect from the study using time spent in general education as a continuous variable.

**Figure 15:**
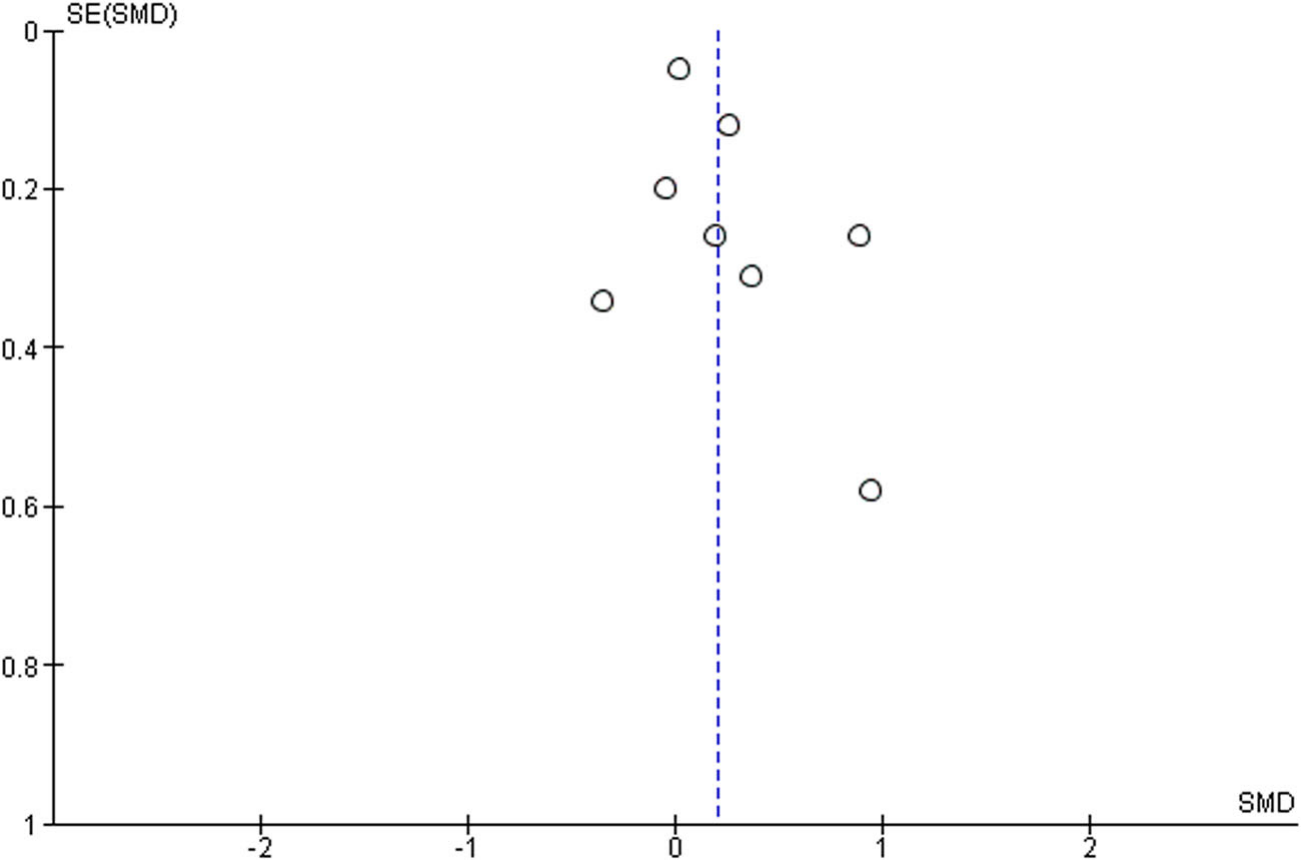
Child overall psychosocial adjustment

**Figure 16:**
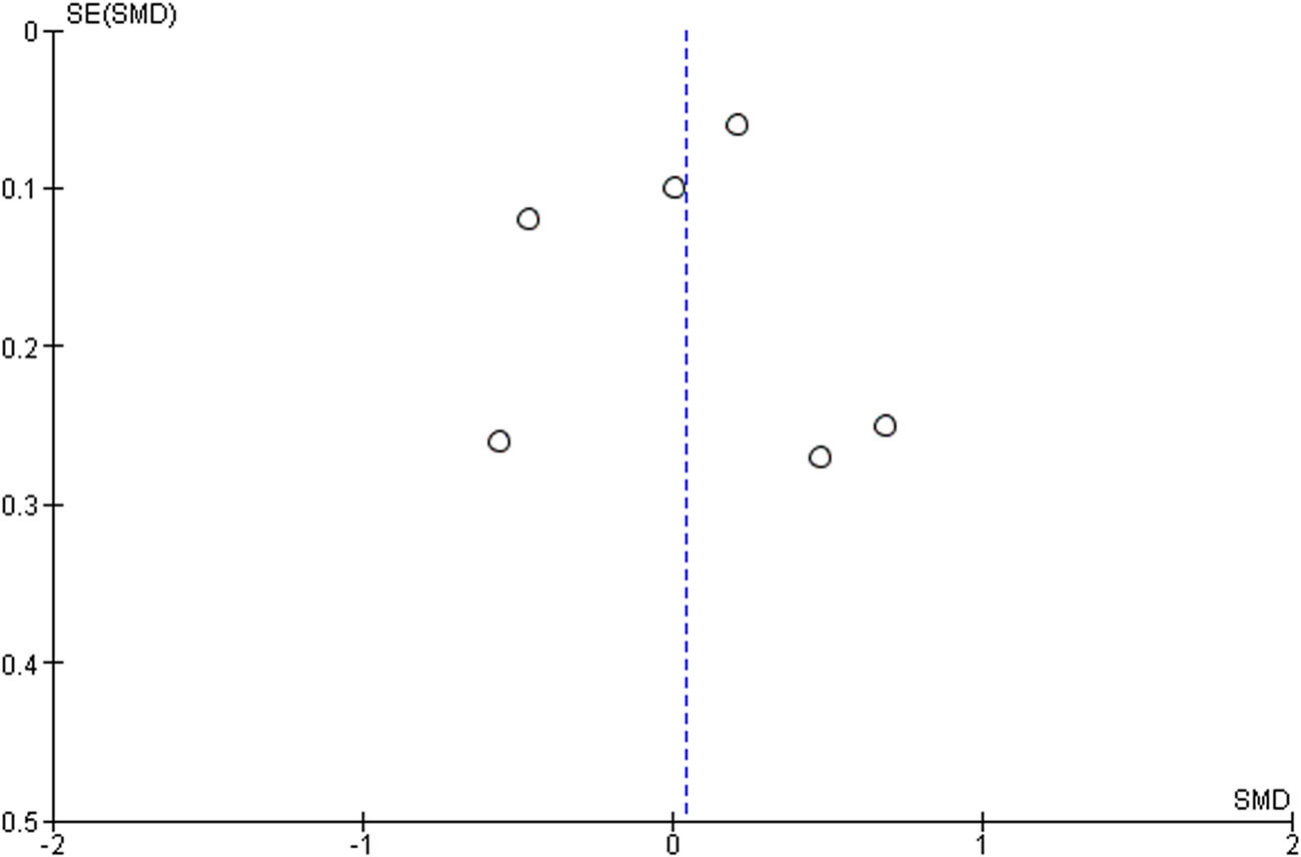
Language and literacy

**Figure 17:**
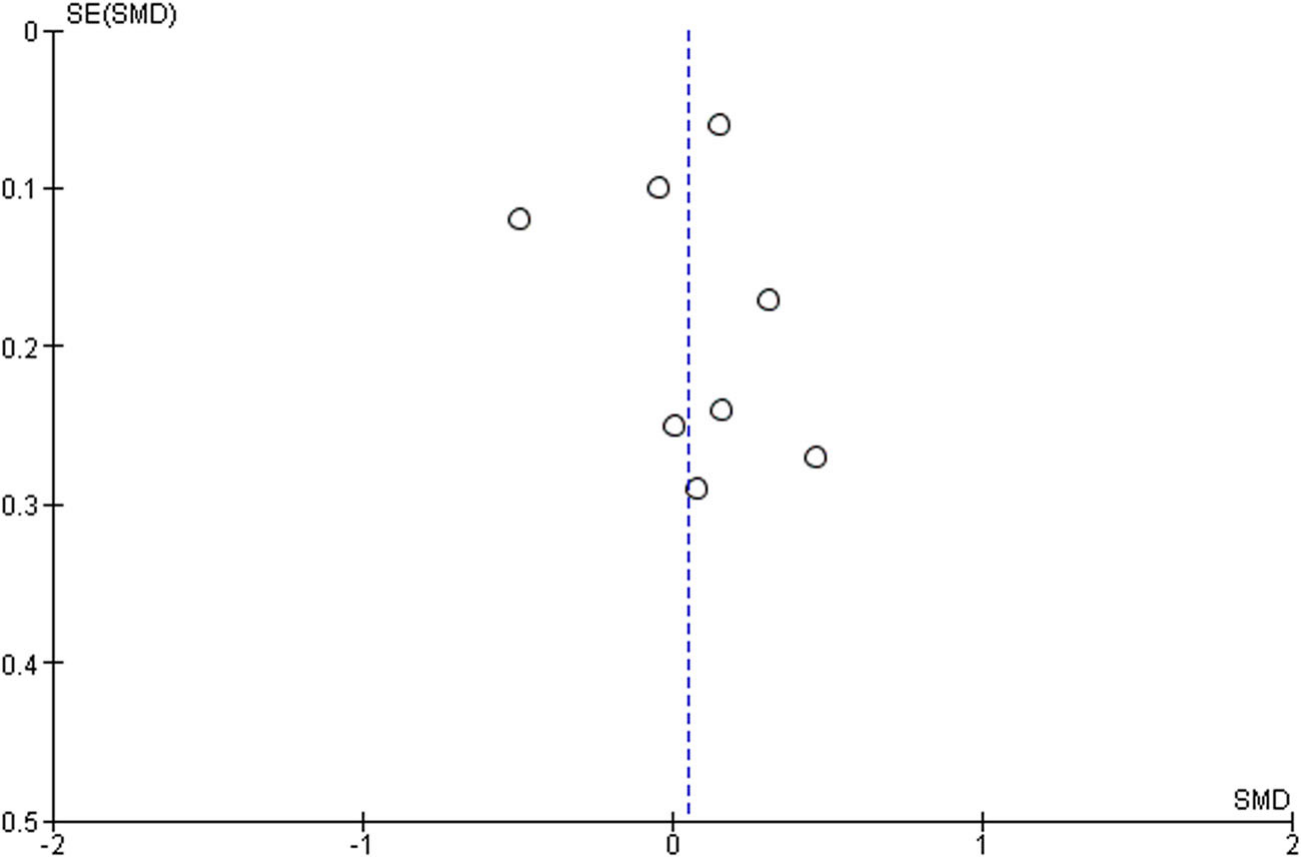
Math

In summary, the conclusions of the main syntheses do not change.

#### 5.4.8 Reporting bias

We constructed funnel plots for all meta‐analyses (e.g., outcomes: child overall psychosocial adjustment, language and literacy and math). The funnel plots were visually examined and there were no striking imbalances (Figures 15, 16, 17).

## 6 DISCUSSION

### 6.1 Summary of main results

Based on the 15 studies, which could be used in the data synthesis, three meta‐analyses were conducted on the outcomes overall psychosocial adjustment, language/literacy, and math. Overall, there were too few studies included in any of the meta‐analyses and the degree of heterogeneity was too high in order for us to draw any conclusion concerning the effectiveness of inclusion. At most, the results from eight studies could be pooled in a single meta‐analysis. All the meta‐analyses showed a positive weighted average and none of them were statistically significant. Heterogeneity was present in all analyses and 95% prediction intervals were wide reflecting that the distribution of effect sizes spanned both large positive and large negative effect sizes.

#### 6.1.1 Subgroup analysis

Single‐factor subgroup analyses were carried out where possible for the moderators: type of disability (only for the outcome child overall psychosocial adjustment); mean age (only for the outcomes: child overall psychosocial adjustment and math) and finally co‐teaching (only for the outcome math), but the evidence was inconclusive as to whether any of these moderators relate to outcomes.

### 6.2 Overall completeness and applicability of evidence

In this review, we included a total of 15 studies in the data‐synthesis. This number is relatively low compared to the large number of studies (94) meeting the inclusion criteria. The reduction was mainly due to the remaining studies being judged to be in critically high risk of bias (mainly confounding bias) and, in accordance with the protocol, we excluded these from the data synthesis on the basis that they would be more likely to mislead than inform on the size of the effect of the intervention. If all the 94 studies had provided an effect estimate with a lower risk of bias, the final list of useable studies in the data synthesis would have been larger which, in turn, would have provided a more robust literature on which to base conclusions.

In total, samples from 19 countries were represented by the 94 studies meeting the inclusion criteria. The 15 studies used in the data synthesis covered only nine countries (Belgium, Czech Republic, Denmark, Finland, Germany, the Netherlands, Switzerland, UK, and USA). The more narrow geographical coverage may limit the applicability of the evidence.

We analysed all available child outcomes. However, the small number of studies reporting on the same outcomes makes us reluctant to draw any firm conclusions, as only between six and eight studies could be pooled in a meta‐analysis. Heterogeneity was present in all analyses and 95% prediction intervals were wide, reflecting that the distribution of effect sizes spanned both large positive and large negative effect sizes. The small number of studies unfortunately precluded a thorough investigation of heterogeneity and the impact of potential moderators, and thus more research is needed.

As can be seen from Table 5, a high proportion of the studies used in the meta‐analyses are based on populations of children in which children with more severe disabilities or special needs were excluded from the analysis. This selection was typically done to ensure comparability between children placed in inclusive settings (treatment) and control children placed in segregated special education. This selection practice reflects the fact that in many school systems, placement of children with special needs is dependent on assessments of symptom severity, with an increased tendency for children with severe and pervasive difficulties to be placed in segregated special education and children with mild or moderate difficulties to be placed in inclusive settings. Therefore, estimating the effects of inclusion based on comparisons between children placed in inclusion vis a vis children in segregated special education is all but straightforward; and while excluding special education children with the most severe disabilities from the analytical sample may be a necessary step to ensure comparability in individual studies, it means that the current review has limited applicability when it comes to the placement conditions of children with the most severe types of special needs or disabilities. This is an unfortunate limitation given the need for more knowledge regarding the effects of educational placement for all children with SEN, regardless of disability type or symptom severity.

We found no indication of publication bias.

Findings from the present review are relevant to policy and decision‐makers as well as to educational placement agencies, as they emphasise the need to base a child's educational placement on an individual assessment of the child's needs. The fact that there were no studies reporting on the long term effects of inclusion is perhaps not surprising as longitudinal research is costly and difficult to carry out. However, based on the theoretical hypothesis that inclusive placement decreases stigma, social isolation and marginalisation of children with special needs, it is possible that these effects do not manifest themselves while the child is still in school, and thus more research should explore this issue further, by including follow‐up measurements of outcomes into adulthood.

### 6.3 Quality of the evidence

The overall methodological quality of the included studies was very low, no experimental studies in which children were randomly assigned to intervention and control conditions were identified. Overall the risk of bias in the included non‐randomised studies was high. We examined the risk of bias using the model ROBINS–I, developed by members of the Cochrane Bias Methods Group and the Cochrane Non‐Randomised Studies Methods Group (Sterne et al., 2016) for non‐randomised studies. Among the included non‐randomised studies, only 15 of 94 studies were not rated critical risk of bias, and of these 15 studies not rated critical risk of bias, only one study had an overall moderate risk of bias, while the remaining 14 studies were judged to be in serious risk of bias. The level ‘critical’ means: the study (outcome) is too problematic in this domain to provide any useful evidence on the effects of intervention, and it is excluded from the data synthesis.

The quality of the evidence in this review was enhanced by excluding studies assessed to be at critical risk of bias using the ROBINS–I tool from the data synthesis. We believe this process excluded those studies that are more likely to mislead than inform.

Furthermore, we performed a number of sensitivity analyses for each outcome to check whether the obtained results were robust across methodological quality, carrying out sensitivity analyses for the confounding, selection, classification, measurement, and reporting risk of bias items of the risk of bias checklists. We examined the robustness of our conclusions when we excluded studies with a serious risk of bias assessment. We further examined the robustness of our conclusions when we increased the reported effects from a one standard deviation increase in time spent in general education to an increase of two standard deviations increase in time spent in general education in the study using time spent in general education as a continuous variable. The overall conclusions did not change.

Overall, in all meta‐analyses, there was a moderate amount of heterogeneity between studies and inconsistency in the direction and magnitude of effects. Some effects favoured inclusion and some effects favoured segregated placements.

### 6.4 Potential biases in the review process

We performed a comprehensive electronic database search, combined with grey literature searching, and hand searching of key journals. All citations were screened by two independent screeners from the review team, and one review author (NTD) assessed all included studies against inclusion criteria.

We believe that all the publicly available studies on the effects of inclusion for children with special needs in the OECD countries published after 2000 were identified during the review process. However, 23 references were not obtained in full text.

We were unable to comment on the possibility of publication bias as at most eight studies were included in the same meta‐analysis. Thus, we cannot rule out that there are still some missing studies.

We believe that there are no other potential biases in the review process as two members of the review team (NTD, ANBO, TF, MWK, MHC) independently coded the included studies. Any disagreements were resolved by discussion. Further, decisions about the inclusion of studies were made by two members of the review team (MWK, MHC) and at least one review author (ANBO, TF, NTD). Assessment of study quality and numeric data extraction was made by one review author (TF/NTD/ANBO) and was checked by a second review author.

Members of the review team at VIVE Campbell were: Research assistants: Malene Wallach Kildemoes and Maluhs Haulund Christensen.

### 6.5 Agreements and disagreements with other studies or reviews

Lindsay (2007) provides a narrative review of the effectiveness of inclusive education for students with SEN. The review provides a historical overview of the vast literature before 2000 and a search of studies published 2001‐2005 in eight journals on special education. The search identified 1373 studies and points to the fact that only 1% of the identified papers were comparative outcome studies. The review concludes that there is a lack of evidence for the effectiveness of inclusion and argues that where evidence does exist, the balance is only marginally positive (Lindsay, 2007). Our findings are very similar to the findings from Lindsay (2007) and to all previous systematic reviews and meta‐analyses, suggesting that even though the number of published studies has grown a lot in the past two decades, the proportion of comparative outcome studies in this field is extremely low, and even when a control group is used, the vast majority of studies are still in serious risk of confounding bias due to the fact that educational placement is not random. To the contrary, educational placement is most often based on an assessment of the individual child's needs, and at the moment this seems like the most ethically sound choice since the evidence base on the effects of inclusion to this day provides little guidance.

## 7 AUTHORS' CONCLUSIONS

### 7.1 Implications for practice

Based on the findings from the present review, it is clear that the quantitative evidence on the effects of inclusion does not suggest that placing children with special needs in inclusive settings is by default superior to placement in traditional segregated special educational settings on measures of children's psychosocial adjustment and learning, rather results should be interpreted with caution. However, we must emphasise that this absence of evidence is not evidence of absence. Heterogeneity was present in all analyses and 95% prediction intervals were wide, reflecting that the distribution of effect sizes spanned both large positive and large negative effect sizes. Further, the possibilities of investigating the impact of potential moderators was limited, and the moderator analyses were inconclusive due to the low number of studies included in each meta‐analysis and due to limited reporting of and variability in the potential moderators.

It is possible that future focused primary studies or systematic reviews may be able to identify significant positive or negative effects for children with certain types of special needs or that specific models of inclusion may be more effective than others at increasing academic achievement, psychosocial adjustment and wellbeing in children with special needs. At the moment, the current evidence base suggests that one size does not fit all, and there is only limited contemporary evidence on what works for whom in special education when it comes to comparing inclusion to segregated placement for subgroups of children with special needs. Furthermore, as stated in the description of included studies in the review, 11 studies were based on child populations in which children with more severe disabilities were excluded from the analysis. This challenges the generalisability of the findings from the present review, and thus there is limited evidence on the effects of inclusion for children with more severe disabilities. Similarly, very few studies contained detailed descriptions of the resources available to children within both inclusive and segregated settings. This constitutes a serious flaw in the evidence base, as it is theoretically possible that the effects of inclusion are dependent upon the resources available to the children within the educational setting.

Therefore, the choice of educational placement should continue to be based on an individual assessment of the child and detailed knowledge of the specific local inclusion model available. As is evident from the included studies in this review as well as in findings from previous reviews, the scientific literature on the effects of inclusion is vast and comes from numerous countries (Lindsay, 2007; Madden, 1983; Ruijs, 2009), and while it is not possible to draw any definitive conclusions regarding the international overall effects of inclusion, practitioners may find useful locally relevant contemporary knowledge by reading some of the included studies within the present review or by exploring the qualitative research literature.

### 7.2 Implications for research

The overall methodological quality of the included studies was very low, no experimental studies in which children were randomly assigned to intervention and control conditions were found. The 15 studies which could be used in the meta‐analyses were all except for one judged to be in serious risk of bias. The risk of bias assessment of the included studies suggests that the risk of confounding bias is a serious problem in almost all available studies. This finding is perhaps not surprising and reflects the fact that educational placement is very seldom random and most often reflects an assessment of the severity of the SEN. Based on this finding, it could be argued that the overall direction of bias favours inclusion, as most of the included studies within the review described school placement procedures in which children with more severe special needs were more likely to be placed in segregated settings.

Unfortunately, within the present review, it was not possible to test the potential moderating effects of severity of SEN across included studies. The participating children had very diverse difficulties and diagnoses ranging from children who only had a specific learning disability to children with multiple severe physical and mental disabilities. However, based on the descriptions of educational placement procedures in the included primary studies, it is clear that in many cases inclusive settings were not considered appropriate for children with the most severe disabilities, and in other cases children with more severe disabilities were excluded from the analyses to ensure balance on potential confounders between intervention and control groups. Thus, findings from the present review might have been different if all children from across the ranges of severity of disability had been included. Within the included studies in the review, the descriptions of the resources available to the children in the included and segregated settings were limited, meaning that in many studies it was unclear if there was co‐teaching or additional personnel within the inclusive classrooms or if children with specific disabilities had access to technology that might enable them to compensate for their disabilities. This needs to be explored further in future studies, as it is theoretically possible that the effects of inclusion are dependent upon the resources available within both the segregated and the inclusive setting.

From a scientific perspective, the evidence base would be strengthened by carrying out randomised studies. However, there are a number of ethical concerns, which need to be addressed. First, it would be important to ensure that the inclusive setting is fitted with sufficient resources and personnel to meet the needs of children with special needs, and thus inclusive education should not become simply a way of reducing the costs of special education. Second, it would be important to conduct an individual assessment of eligibility of the child before randomisation, to ensure that there are no individual needs of the specific child which can only be met in either an inclusive or a segregated educational placement. Third, participation in a randomised trial should be based on informed consent from the parents, and thus it may not be feasible to conduct randomised studies within this field.

Results of the meta‐analyses do not suggest on average any sizeable positive or negative effects of inclusion on children's academic achievement in language, literacy, and math, or in the overall psychosocial adjustment of children. The average point estimates did favour inclusion, though small and not statistically significant, and heterogeneity was present in all analyses and there was inconsistency in direction and magnitude of the effect sizes.

This finding is very similar to the results of previous systematic reviews and meta‐analyses, which include studies published before 2000, and thus although the number of studies in the current meta‐analyses is rather limited, it can be concluded that it is very unlikely that inclusion in general increases learning and psychosocial adjustment for children with special needs. Rather, the results may be seen to suggest that for some children with special needs, segregated placement may be the better educational choice—at least when compared with the current local models of inclusion. Future research should thus aim to explore the effects of different kinds of inclusive education for different kinds of children with special needs, to expand the knowledge base on what works for whom.

In this review, none of the studies which could be used in the meta‐analyses reported usable follow‐up outcomes and this finding is also similar to reviews published before 2000. Theoretically, it is possible that potential positive effects of inclusion emerge at a later stage in the child's life, and thus it would be very useful for future research to include time points past the end of the intervention. More specifically, it would be useful to explore outcomes such as social skills, social integration, self‐perception, marginalisation and employment to fully test the ideological and theoretical hypotheses that inclusive education may protect the child from the effects of stigma and lead to more social integration and less marginalisation or social isolation in adulthood.

## CONTRIBUTIONS OF AUTHORS

- Content:
  Nina T. Dalgaard is a psychologist, PhD Nina has previously worked as both an educational psychologist within a primary school setting and as a clinical child psychologist and thus has knowledge about the socio‐emotional and cognitive development of children.
  Anja Bondebjerg holds a Master's degree in Sociology and has worked extensively with systematic reviews and research mappings in the fields of education and early childhood education and care. She is knowledgeable regarding the structure and process of conducting systematic reviews.
- Systematic review methods:
  Trine Filges, PhD (economics) is an experienced systematic reviewer and methodologist, having completed a number of systematic reviews in social welfare topic areas as well as in the field of education. Trine has published nineteen Campbell Systematic reviews, is currently the lead reviewer on one Campbell Systematic Review, further involved as a reviewer in three Campbell Systematic Reviews and has published systematic and meta‐analytic reviews in high‐impact journals. Trine's fields of expertise are systematic review methods and statistical analysis; and she contributed to and supervised the quantitative data extraction, methodological quality appraisal and meta‐analysis.
  Anja Bondebjerg (please see description above)
- Statistical analysis:
  Trine Filges (please see description above)
- Information retrieval:

Bjørn C. A. Viinholt (information specialist) has 4 years of experience in developing and writing systematic reviews. As a part of undertaking systematic reviews, Bjørn has experience in developing systematic search strategies and processes of reference management. Bjørn contributed with assisting and development of the systematic search strategy, executing the searches, and assisted with reference management and grey literature searches.

## DIFFERENCES BETWEEN PROTOCOL AND REVIEW

We calculated prediction intervals to ease interpretation of the impact of heterogeneity, although this was not planned at the protocol phase.

## PUBLISHED NOTES

Characteristics of studies

Characteristics of included studies Risk of bias Tables are available online as Supporting Information files [Link], [Link], [Link].

**Characteristics of excluded studies**

**Table:** 

Bishop, 2017	
**Reason for exclusion**	Study design: before and after
Blanda‐Holtzberg, 2004	
**Reason for exclusion**	Study design: Qualitative
de Graaf,	
**Reason for exclusion**	Reading scores are measured by parent report ‐ not a relevant outcome
de Graaf, 2013/01	
**Reason for exclusion**	Children with Downs syndrome and outcome is parent evaluation of their reading, writing and math skills
Doğru, 2007/11	
**Reason for exclusion**	Outcome is emotion recognition, which is not relevant to the present review
Emery, 2009/01	
**Reason for exclusion**	Wrong design: students serve as their own control
Ewing, 2009/01	
**Reason for exclusion**	Compare four groups: Entered Special Education, Exited Special Education, Always in Special Education and Always in General Education (no disabilities). No analysis of effects of inclusion
Fang, 2018	
**Reason for exclusion**	Participants are from Taiwan
Farmer, 2000/07	
**Reason for exclusion**	Students from one special school compared to children from two general education schools
Farrell 2007/09	
**Reason for exclusion**	No analysis at individual level, school and local authority levels only
Feldman 2019/06	
**Reason for exclusion**	It is about predicting change in student–teacher conflict and closeness using among others class placement (mainstream or special education) as a moderator
Finnvold, 2018/01	
**Reason for exclusion**	Outcome is not relevant to the present review: Seeing friends or participating in after‐school formal group activities, not standardised
Floyd, 2014/01	
**Reason for exclusion**	Schools are the unit of analysis, not students, as seen in the following statement: ‘The population for this study included nine charter (full inclusion) and nine traditional public schools (self‐contained) in Michigan that had at least two years of standardised testing data for the students with disabilities subgroup’
Flynn, 2013	
**Reason for exclusion**	No comparison of inclusion vs segregated placement, the study examines students' movement in and out of special education and predictors for later special education placement.
Gaddis, 2005	
**Reason for exclusion**	Two schools are treated (two different inclusion programmes) A third school in which no inclusion program has been implemented served as a control group
Gasteiger‐Klicpera, 2013/07	
**Reason for exclusion**	Wrong outcome: parental attitudes and experiences are not relevant outcomes in the present review
Gorges, 2018/01	
**Reason for exclusion**	Investigates the reciprocal effects between self‐concepts and performance of SEN‐L students with inclusive versus exclusive setting as a moderator
Haller, 2007	
**Reason for exclusion**	No control group
***Hanushek***	
**Reason for exclusion**	The primary analysis is about the effect of special education (eg. there is no distinguishing between programmes mainstreaming and not mainstreaming)
Harper, 2002/07	
**Reason for exclusion**	Preschool intervention
Harrington, 2011/01	
**Reason for exclusion**	Uses an IV model
Hatch, 2014	
**Reason for exclusion**	Only one homeroom class in each condition
Hestenes, 2008/07	
**Reason for exclusion**	Participants are in preschool
Howard‐Parker, 2006	
**Reason for exclusion**	Only one class compared to one other class (p. 55 and 58)
Juettemeyer, 2013	
**Reason for exclusion**	Comparison of cotaught classes and general education classes
Kersey, 2013	
**Reason for exclusion**	Compares co‐teaching with regular class rooms
Kim, 2018/03	
**Reason for exclusion**	This study examines early predictors of and changes in school‐age academic achievement and class placement in children referred for autism spectrum disorder (ASD) at age 2. Class placement is adressed descriptively in the present study and no causality can be inferred between changes in class placement and academic development in adolescents with ASD from these results. There is an online table showing Characteristics (including relevant outcomes such as reading and math scores) of children who maintained or lost access to typical peers (i.e., inclusion throughout (the ages 9–18) and transition from inclusion to special class somewhere between the age of 9 and 18, exact time not reported). The numbers are 26 and 30, thus there are 55 who remained in special education classes not included in the table.
McPhillips, 2007	
**Reason for exclusion**	Case study of different schools, no effect study
Nikolic, 2019	
**Reason for exclusion**	Serbia is not a member of OECD
Nolan, 2020	
**Reason for exclusion**	Only schools are analysed, not students
Ocque Karen, 2017	
**Reason for exclusion**	compares integrated cotaught (ICT) classrooms and with no ICT classrooms (regular class rooms for students without special needs)
Olsson, 2017/09	
**Reason for exclusion**	Wrong outcome: The utilisation of habilitation services outside school
Olsson, 2020/02	
**Reason for exclusion**	Wrong outcome: The utilisation of habilitation services outside school
Pandey, 2018/01	
**Reason for exclusion**	India is not in the OECD
Pant, 2016/01	
**Reason for exclusion**	India is not in the OECD
Salgado, 2001	
**Reason for exclusion**	No effects of inclusion for special needs students, comparison is students without disabilities
Savitz, 2005/03	
**Reason for exclusion**	Outcome is not relevant to the present review
Saylor, 2017/01	
**Reason for exclusion**	No students are in special/segregated placement
Schulte, 2004/06	
**Reason for exclusion**	The purpose of this study was to use school results on a large‐scale, high‐stakes reading test across several years to illustrate the complexities and issues involved in reporting school‐based special education outcomes. Test scores from six elementary schools in one school district in North Carolina were used to examine three different ways of reporting school‐based reading outcomes for students in special education in terms of their ability to provide meaningful, valid information about a school's functioning with students in special education: (a) percentage of students in special education reaching grade‐level proficiency in reading each year, (b) percentage of students in special education exceeding expected growth in reading each year, and (c) longitudinal growth and percentage of students reaching grade‐level proficiency in fifth grade. This, this study is not concerned with the effects of inclusion versus special education and should therefore be late‐stage excluded.
Sion‐Johnson, 2003	
**Reason for exclusion**	This study investigated the attitudes of regular students and students classified with LD, ED, and EMD. The Survey of Reading Attitudes (SRA) and the Fennema‐Sherman Mathematics Attitude Scales (FSAS‐R) were administered to 215 students in grades 4, 5, 6, and 7 of two school districts in the southeastern United States. The present study distinguishes between special education groups (in terms of diagnosis/type of diffulty, i.e., LD, ED, and EMD) and regular education students. So placement here means special education for different types of disabilities, not placement in the sense of more or less restrictive environment as we are interested in. The differences explored in the study are either between special education students and regular students without disabilities or between different types of special education students. There is no investigation of differences related to the degree of inclusiveness of placement. Therefore, this study is not relevant to our review.
Siperstein 2011/05	
**Reason for exclusion**	This study compares three subgroups: children with ED receiving special education in low‐income schools, children with ED receiving special education in high‐income schools, and children not receiving special education services but who were considered high risk for ED. The study does not compare the effects of different types of special education placements (more or less inclusive), but rather deals with differences pertaining to school income, being at at risk versus having the diagnosis, and receiving different services (such as counselling, speech therapy, etc.).
Son, 2012	
**Reason for exclusion**	This longitudinal study analysed three waves of data/three cohorts of children from the Pre‐Elementary Education Longitudinal Study (PEELS) data (N = 1,268). The study examined the prevalence, nature, and pathways between child characteristics, family factors, school factors at Wave 1, peer‐relation difficulties at Wave 2, and peer victimisation at Wave 3. The study used a nationally representative, longitudinal survey sample of 1,268 children from 258 school districts, ages 3 through 5, who were receiving special education services in the 2003–2004 academic year. The participating children are mainly in pre‐elementary which is not the right setting for this review. Furthermore, comparions are not made between clearly defined groups of children placed in inclusion versus special education.

## DATA AND ANALYSES

1 Child outcomes

**Table:** 

Outcome or Subgroup	Studies	Participants	Statistical Method	Effect Estimate
1.1 Child overall psychosocial	8	Std. Mean Difference (IV, Random, 95% CI)		0.20 [−0.01, 0.42]
1.2 Language and literacy	6	Std. Mean Difference (IV, Random, 95% CI)		0.04 [−0.27, 0.35]
1.3 Math	8	Std. Mean Difference (IV, Random, 95% CI)		0.05 [−0.16, 0.26]
1.4 Child overall psychosocial Autism single factor subgroup	8	Std. Mean Difference (IV, Random, 95% CI)		Subtotals only
1.4.1 Autism	2	Std. Mean Difference (IV, Random, 95% CI)		−0.12 [−0.46, 0.22]
1.4.2 Not autism	6	Std. Mean Difference (IV, Random, 95% CI)		0.32 [0.05, 0.59]
1.5 Child overall psychosocial Physical disabilities single factor subgroup	8	Std. Mean Difference (IV, Random, 95% CI)		Subtotals only
1.5.1 Physical disabilities	2	Std. Mean Difference (IV, Random, 95% CI)		0.06 [−0.16, 0.28]
1.5.2 Non‐physical disabilities	6	Std. Mean Difference (IV, Random, 95% CI)		0.26 [−0.06, 0.57]
1.6 Child overall psychosocial Age 15 single factor subgroup	8	Std. Mean Difference (IV, Random, 95% CI)		Subtotals only
1.6.1 Age 15 or higher	3	Std. Mean Difference (IV, Random, 95% CI)		0.47 [0.08, 0.86]
1.6.2 Age less than 15	5	Std. Mean Difference (IV, Random, 95% CI)		0.02 [−0.10, 0.15]
1.7 Child overall psychosocial Age 10 single factor subgroup	8	Std. Mean Difference (IV, Random, 95% CI)		Subtotals only
1.7.1 Age 10 or higher	6	Std. Mean Difference (IV, Random, 95% CI)		0.27 [−0.02, 0.56]
1.7.2 Age less than 10	2	Std. Mean Difference (IV, Random, 95% CI)		0.05 [−0.26, 0.36]
1.8 Math Co‐taught single factor subgroup	8	Std. Mean Difference (IV, Random, 95% CI)		Subtotals only
1.8.1 Co‐taught	2	Std. Mean Difference (IV, Random, 95% CI)		0.35 [0.07, 0.63]
1.8.2 Not co‐taught	6	Std. Mean Difference (IV, Random, 95% CI)		−0.04 [−0.28, 0.20]
1.9 Math Age 15 single factor subgroup	8	Std. Mean Difference (IV, Random, 95% CI)		Subtotals only
1.9.1 Age 15 or higher	2	Std. Mean Difference (IV, Random, 95% CI)		−0.10 [−0.88, 0.68]
1.9.2 Age less than 15	6	Std. Mean Difference (IV, Random, 95% CI)		0.11 [0.02, 0.20]

## SOURCES OF SUPPORT

**Internal sources**

• No sources of support provided

**External sources**

- No sources of support provided

## Supporting information

Supporting information.

Supporting information.

Supporting information.

Supporting information.

## Footnotes

^1^
The review was retrieved 02.10.2019 from: https://files.eric.ed.gov/fulltext/ED519296.pdf.

^2^
For more information on the IDEA Act disability categories, go to: https://sites.ed.gov/idea/regs/b/a/300.8 (the US Department of Education's Individuals with Disabilities Education Act (IDEA) website).
