# Supplementary material for: The effects of inclusion on academic achievement, socioemotional development and wellbeing of children with special educational needs
Source: Campbell Syst Rev. 2022 Dec 7;18(4):e1291. doi: 10.1002/cl2.1291 (PMC9727566; doi:10.1002/cl2.1291)
Supplement: Supplementary file 1 — Supporting information. [file CL2-18-e1291-s003.docx]

# Appendices

## 1 Electronic Searches I

Academic Search Premier. Searched 07/04/2021. Limiters - Published Date: 20000101- . Search modes - Boolean/Phrase. Interface - EBSCOhost Research Databases. Search Screen - Basic Search.

| **Search** | **Search Terms** | **Results** |
| --- | --- | --- |
| S13 | S4 AND S8 AND S12 | 2,912 |
| S12 | S9 OR S10 OR S11 | 13,025,960 |
| S11 | DE "EFFECT sizes (Statistics)" OR DE CONTROL groups" OR DE "Experimental Groups" OR DE "EXPERIMENTS" OR DE "MATCHED groups" OR DE "Randomized Controlled Trials" | 9,670 |
| S10 | AB (effect* OR trial* OR experiment* OR control* OR random* OR impact* OR compar* OR difference*) | 12,446,676 |
| S9 | TI (effect* OR trial* OR experiment* OR control* OR random* OR impact* OR compar* OR difference*) | 3,069,133 |
| S8 | S5 OR S6 OR S7 | 1,294,675 |
| S7 | DE "SCHOOL grade placement" OR DE "INCLUSIVE education" OR DE "MAINSTREAMING in special education" | 5,722 |
| S6 | AB (integrat* OR immers* OR inclus* OR mainstream* OR placement*) | 1,227,948 |
| S5 | TI (integrat* OR immers* OR inclus* OR mainstream* OR placement*) | 227,396 |
| S4 | (S1 AND S2) OR S3 | 52,997 |
| S3 | DE "SPECIAL needs students" | 1,443 |
| S2 | TI (need* OR special* OR additional*) OR AB ((special* OR additional* OR educational*) N5 (need*)) | 324,080 |
| S1 | TI (student* OR pupil* OR child* OR youth* OR young*) OR AB (student* OR pupil* OR child* OR youth* OR young*) | 2,575,492 |

ECONLIT. Search Performed 08/04/2021. Interface - EBSCOhost Research Databases.
Search Screen - Advanced Search. Limiters - Published Date: 20000101-.  Search modes - Boolean/Phrase.

| **Search** | **Search Terms** | **Results** |
| --- | --- | --- |
| S10 | S3 AND S6 AND S9 | 34 |
| S9 | S7 OR S8 | 630,149 |
| S8 | AB (effect* OR trial* OR experiment* OR control* OR random* OR impact* OR compar* OR difference*) | 555,137 |
| S7 | TI (effect* OR trial* OR experiment* OR control* OR random* OR impact* OR compar* OR difference*) | 191,300 |
| S6 | S4 OR S5 | 78,242 |
| S5 | AB (integrat* OR immers* OR inclus* OR mainstream* OR placement*) | 66,563 |
| S4 | TI (integrat* OR immers* OR inclus* OR mainstream* OR placement*) | 23,245 |
| S3 | S1 AND S2 | 828 |
| S2 | TI (need* OR special* OR additional*) OR AB ((special* OR additional* OR educational*) N5 (need*)) | 16,199 |
| S1 | TI (student* OR pupil* OR child* OR youth* OR young*) OR AB (student* OR pupil* OR child* OR youth* OR young*) | 66,661 |

ERIC. Search performed 07/04/2021.  Limiters - Date Published: 20000101- . Search modes - Boolean/Phrase. Interface - EBSCOhost Research Databases. Search Screen - Advanced Search.

| **Search** | **Search Terms** | **Results** |
| --- | --- | --- |
| S13 | S4 AND S8 AND S12 | 3,338 |
| S12 | S9 OR S10 OR S11 | 727,679 |
| S11 | DE "Effect Size" OR DE "Control Groups" OR DE "Experimental Groups" OR DE "Experiments" OR DE "Matched Groups" OR DE "Quasiexperimental Design" OR DE "Randomized Controlled Trials" OR DE "Comparative Testing" | 29,947 |
| S10 | AB (effect* OR trial* OR experiment* OR control* OR random* OR impact* OR compar* OR difference*) | 687,960 |
| S9 | TI (effect* OR trial* OR experiment* OR control* OR random* OR impact* OR compar* OR difference*) | 173,157 |
| S8 | S5 OR S6 OR S7 | 166,966 |
| S7 | DE "Placement" OR DE "Academic Accommodations (Disabilities)" OR DE "Inclusion" OR DE "Mainstreaming" OR DE "Student Placement" | 27,753 |
| S6 | AB (integrat* OR immers* OR inclus* OR mainstream* OR placement*) | 150,636 |
| S5 | TI (integrat* OR immers* OR inclus* OR mainstream* OR placement*) | 35,892 |
| S4 | (S1 AND S2) OR S3 | 51,296 |
| S3 | DE ("Special Needs Students") | 9,448 |
| S2 | TI (need* OR special* OR additional*) OR AB ((special* OR additional* OR educational*) N5 (need*)) | 72,060 |
| S1 | TI (student* OR pupil* OR child* OR youth* OR young*) OR AB (student* OR pupil* OR child* OR youth* OR young*) | 992,628 |

PsycINFO. Search Performed 07/04/2021. Interface - EBSCOhost Research Databases. Search Screen - Advanced Search. Limiters - Published Date: 20000101- .Search modes - Boolean/Phrase.

| **Search** | **Search Terms** | **Results** |
| --- | --- | --- |
| S13 | S4 AND S8 AND S12 | 3,182 |
| S12 | S9 OR S10 OR S11 | 2,830,386 |
| S11 | DE "Effect Size" OR DE "Control Groups" OR DE "Experimental Groups" OR DE "Experiments" OR DE "Matched Groups" OR DE "Quasiexperimental Design" OR DE "Randomized Controlled Trials" OR DE "Comparative Testing" | 1,235 |
| S10 | AB (effect* OR trial* OR experiment* OR control* OR random* OR impact* OR compar* OR difference*) | 2,696,492 |
| S9 | TI (effect* OR trial* OR experiment* OR control* OR random* OR impact* OR compar* OR difference*) | 739,679 |
| S8 | S5 OR S6 OR S7 | 352,254 |
| S7 | DE "Placement" OR DE "Academic Accommodations (Disabilities)" OR DE "Inclusion" OR DE "Mainstreaming" OR DE "Student Placement" | 965 |
| S6 | AB (integrat* OR immers* OR inclus* OR mainstream* OR placement*) | 336,471 |
| S5 | TI (integrat* OR immers* OR inclus* OR mainstream* OR placement*) | 61,274 |
| S4 | (S1 AND S2) OR S3 | 39,663 |
| S3 | DE ("Special Needs Students") | 51,020 |
| S2 | TI (need* OR special* OR additional*) OR AB ((special* OR additional* OR educational*) N5 (need*)) | 94,319 |
| S1 | TI (student* OR pupil* OR child* OR youth* OR young*) OR AB (student* OR pupil* OR child* OR youth* OR young*) | 1,371,893 |

SocIndex. Search performed 07/04 2021. Limiters - Date of Publication: 20000101- . Search modes - Boolean/Phrase. Interface - EBSCOhost Research Databases. Search Screen - Advanced Search.

| **Search** | **Search Terms** | **Results** |
| --- | --- | --- |
| S13 | S4 AND S8 AND S12 | 551 |
| S12 | S9 OR S10 OR S11 | 828,623 |
| S11 | DE "Randomized Controlled Trials" | 2,614 |
| S10 | AB (effect* OR trial* OR experiment* OR control* OR random* OR impact* OR compar* OR difference*) | 787,127 |
| S9 | TI (effect* OR trial* OR experiment* OR control* OR random* OR impact* OR compar* OR difference*) | 185,887 |
| S8 | S5 OR S6 OR S7 | 122,114 |
| S7 | DE "ABILITY grouping (Education)" OR DE "INCLUSIVE education" OR DE "MAINSTREAMING in special education" OR DE "TRACK system (Education)" | 1,019 |
| S6 | AB (integrat* OR immers* OR inclus* OR mainstream* OR placement*) | 115,095 |
| S5 | TI (integrat* OR immers* OR inclus* OR mainstream* OR placement*) | 23,093 |
| S4 | (S1 AND S2) OR S3 | 13,650 |
| S3 | DE "CHILDREN with disabilities" | 1,870 |
| S2 | TI (need* OR special* OR additional*) OR AB ((special* OR additional* OR educational*) N5 (need*)) | 38,377 |
| S1 | TI (student* OR pupil* OR child* OR youth* OR young*) OR AB (student* OR pupil* OR child* OR youth* OR young*) | 491,181 |

International Bibliography of the Social Sciences (IBSS). Searched through the ProQuest interface. Search performed 08/04/2021.

| **Set** | **Search** | **Results** |
| --- | --- | --- |
| **S14** | [S5 AND S9 AND S13](https://search.proquest.com/recentsearches.recentsearchtabview.recentsearchesgridview.scrolledrecentsearchlist.checkdbssearchlink:rerunsearch/C0331C63EC584CF8PQ/None?site=ibss&t:ac=RecentSearches) | 2,510 |
| **S13** | [S10 OR S11 OR S12](https://search.proquest.com/recentsearches.recentsearchtabview.recentsearchesgridview.scrolledrecentsearchlist.checkdbssearchlink:rerunsearch/41F1C833CEBF4AE4PQ/None?site=ibss&t:ac=RecentSearches) | [1,157,337](https://search.proquest.com/recentsearches.recentsearchtabview.recentsearchesgridview.scrolledrecentsearchlist.checkdbssearchlink_0:rerunsearch/41F1C833CEBF4AE4PQ/None?site=ibss&t:ac=RecentSearches) |
| **S12** | [MAINSUBJECT.EXACT("Clinical trials") OR MAINSUBJECT.EXACT("Experiments")](https://search.proquest.com/recentsearches.recentsearchtabview.recentsearchesgridview.scrolledrecentsearchlist.checkdbssearchlink:rerunsearch/80AE8125FB3F4D16PQ/None?site=ibss&t:ac=RecentSearches) | [13,174](https://search.proquest.com/recentsearches.recentsearchtabview.recentsearchesgridview.scrolledrecentsearchlist.checkdbssearchlink_0:rerunsearch/80AE8125FB3F4D16PQ/None?site=ibss&t:ac=RecentSearches) |
| **S11** | [ab(effect* OR trial* OR experiment* OR control* OR random* OR impact* OR compar* OR difference*)](https://search.proquest.com/recentsearches.recentsearchtabview.recentsearchesgridview.scrolledrecentsearchlist.checkdbssearchlink:rerunsearch/7FB68F62610442FBPQ/None?site=ibss&t:ac=RecentSearches) | [997,865](https://search.proquest.com/recentsearches.recentsearchtabview.recentsearchesgridview.scrolledrecentsearchlist.checkdbssearchlink_0:rerunsearch/7FB68F62610442FBPQ/None?site=ibss&t:ac=RecentSearches) |
| **S10** | [ti(effect* OR trial* OR experiment* OR control* OR random* OR impact* OR compar* OR difference*)](https://search.proquest.com/recentsearches.recentsearchtabview.recentsearchesgridview.scrolledrecentsearchlist.checkdbssearchlink:rerunsearch/D0A277E071048F4PQ/None?site=ibss&t:ac=RecentSearches) | [345,575](https://search.proquest.com/recentsearches.recentsearchtabview.recentsearchesgridview.scrolledrecentsearchlist.checkdbssearchlink_0:rerunsearch/D0A277E071048F4PQ/None?site=ibss&t:ac=RecentSearches) |
| **S9** | [S6 OR S7 OR S8](https://search.proquest.com/recentsearches.recentsearchtabview.recentsearchesgridview.scrolledrecentsearchlist.checkdbssearchlink:rerunsearch/233A25DB7712415FPQ/None?site=ibss&t:ac=RecentSearches) | [172,853](https://search.proquest.com/recentsearches.recentsearchtabview.recentsearchesgridview.scrolledrecentsearchlist.checkdbssearchlink_0:rerunsearch/233A25DB7712415FPQ/None?site=ibss&t:ac=RecentSearches) |
| **S8** | [MAINSUBJECT.EXACT("Child placement") OR MAINSUBJECT.EXACT("Mainstreaming")](https://search.proquest.com/recentsearches.recentsearchtabview.recentsearchesgridview.scrolledrecentsearchlist.checkdbssearchlink:rerunsearch/2B08C61A7B0C4453PQ/None?site=ibss&t:ac=RecentSearches) | [394](https://search.proquest.com/recentsearches.recentsearchtabview.recentsearchesgridview.scrolledrecentsearchlist.checkdbssearchlink_0:rerunsearch/2B08C61A7B0C4453PQ/None?site=ibss&t:ac=RecentSearches) |
| **S7** | [ab(integrat* OR immers* OR inclus* OR mainstream* OR placement*)](https://search.proquest.com/recentsearches.recentsearchtabview.recentsearchesgridview.scrolledrecentsearchlist.checkdbssearchlink:rerunsearch/E8B838D3F87C4984PQ/None?site=ibss&t:ac=RecentSearches) | [156,626](https://search.proquest.com/recentsearches.recentsearchtabview.recentsearchesgridview.scrolledrecentsearchlist.checkdbssearchlink_0:rerunsearch/E8B838D3F87C4984PQ/None?site=ibss&t:ac=RecentSearches) |
| **S6** | [ti(integrat* OR immers* OR inclus* OR mainstream* OR placement*)](https://search.proquest.com/recentsearches.recentsearchtabview.recentsearchesgridview.scrolledrecentsearchlist.checkdbssearchlink:rerunsearch/ED225F4FDFE64A2APQ/None?site=ibss&t:ac=RecentSearches) | [39,322](https://search.proquest.com/recentsearches.recentsearchtabview.recentsearchesgridview.scrolledrecentsearchlist.checkdbssearchlink_0:rerunsearch/ED225F4FDFE64A2APQ/None?site=ibss&t:ac=RecentSearches) |
| **S5** | [(S1 AND S2) OR S3](https://search.proquest.com/recentsearches.recentsearchtabview.recentsearchesgridview.scrolledrecentsearchlist.checkdbssearchlink:rerunsearch/D40D7BB9AE894CD2PQ/None?site=ibss&t:ac=RecentSearches) | [44,094](https://search.proquest.com/recentsearches.recentsearchtabview.recentsearchesgridview.scrolledrecentsearchlist.checkdbssearchlink_0:rerunsearch/D40D7BB9AE894CD2PQ/None?site=ibss&t:ac=RecentSearches) |
| **S3** | [MAINSUBJECT.EXACT("Exceptional children")](https://search.proquest.com/recentsearches.recentsearchtabview.recentsearchesgridview.scrolledrecentsearchlist.checkdbssearchlink:rerunsearch/C237B07E947744C1PQ/None?site=ibss&t:ac=RecentSearches) | [52](https://search.proquest.com/recentsearches.recentsearchtabview.recentsearchesgridview.scrolledrecentsearchlist.checkdbssearchlink_0:rerunsearch/C237B07E947744C1PQ/None?site=ibss&t:ac=RecentSearches) |
| **S2** | [ti(need* OR special* OR additional*) OR ab(need* OR special* OR additional*)](https://search.proquest.com/recentsearches.recentsearchtabview.recentsearchesgridview.scrolledrecentsearchlist.checkdbssearchlink:rerunsearch/B9633502EA3B4326PQ/None?site=ibss&t:ac=RecentSearches) | [320,224](https://search.proquest.com/recentsearches.recentsearchtabview.recentsearchesgridview.scrolledrecentsearchlist.checkdbssearchlink_0:rerunsearch/B9633502EA3B4326PQ/None?site=ibss&t:ac=RecentSearches) |
| **S1** | [ti(student* OR pupil* OR child* OR youth* OR young*) OR ab(student* OR pupil* OR child* OR youth* OR young*)](https://search.proquest.com/recentsearches.recentsearchtabview.recentsearchesgridview.scrolledrecentsearchlist.checkdbssearchlink:rerunsearch/216732B93AF84980PQ/None?site=ibss&t:ac=RecentSearches) | [254,251](https://search.proquest.com/recentsearches.recentsearchtabview.recentsearchesgridview.scrolledrecentsearchlist.checkdbssearchlink_0:rerunsearch/216732B93AF84980PQ/None?site=ibss&t:ac=RecentSearches) |

## 2 Elecronic Searches II

Sociological Abstracts. Searched through the ProQuest interface. Search Performed 08/04/2021.

| set | search | results |
| --- | --- | --- |
| S10 | [S3 AND S6 AND S9](https://search.proquest.com/recentsearches.recentsearchtabview.recentsearchesgridview.scrolledrecentsearchlist.checkdbssearchlink:rerunsearch/CDB50033A7F54C0APQ/None?site=sociologicalabstracts&t:ac=RecentSearches) | [375](https://search.proquest.com/recentsearches.recentsearchtabview.recentsearchesgridview.scrolledrecentsearchlist.checkdbssearchlink_0:rerunsearch/CDB50033A7F54C0APQ/None?site=sociologicalabstracts&t:ac=RecentSearches) |
| S9 | [S7 OR S8](https://search.proquest.com/recentsearches.recentsearchtabview.recentsearchesgridview.scrolledrecentsearchlist.checkdbssearchlink:rerunsearch/75A776FAB37D471APQ/None?site=sociologicalabstracts&t:ac=RecentSearches) | [482,602](https://search.proquest.com/recentsearches.recentsearchtabview.recentsearchesgridview.scrolledrecentsearchlist.checkdbssearchlink_0:rerunsearch/75A776FAB37D471APQ/None?site=sociologicalabstracts&t:ac=RecentSearches) |
| S8 | [MAINSUBJECT.EXACT("Experiments")](https://search.proquest.com/recentsearches.recentsearchtabview.recentsearchesgridview.scrolledrecentsearchlist.checkdbssearchlink:rerunsearch/F8CF3943C40A4D60PQ/None?site=sociologicalabstracts&t:ac=RecentSearches) | [2,243](https://search.proquest.com/recentsearches.recentsearchtabview.recentsearchesgridview.scrolledrecentsearchlist.checkdbssearchlink_0:rerunsearch/F8CF3943C40A4D60PQ/None?site=sociologicalabstracts&t:ac=RecentSearches) |
| S7 | [ti(effect* OR trial* OR experiment* OR control* OR random* OR impact* OR compar* OR difference*) OR ab(effect* OR trial* OR experiment* OR control* OR random* OR impact* OR compar* OR difference*)](https://search.proquest.com/recentsearches.recentsearchtabview.recentsearchesgridview.scrolledrecentsearchlist.checkdbssearchlink:rerunsearch/E3F9AA69F4E047B0PQ/None?site=sociologicalabstracts&t:ac=RecentSearches) | [482,522](https://search.proquest.com/recentsearches.recentsearchtabview.recentsearchesgridview.scrolledrecentsearchlist.checkdbssearchlink_0:rerunsearch/E3F9AA69F4E047B0PQ/None?site=sociologicalabstracts&t:ac=RecentSearches) |
| S6 | [S4 OR S5](https://search.proquest.com/recentsearches.recentsearchtabview.recentsearchesgridview.scrolledrecentsearchlist.checkdbssearchlink:rerunsearch/7F8452F12E5C433EPQ/None?site=sociologicalabstracts&t:ac=RecentSearches) | [99,327](https://search.proquest.com/recentsearches.recentsearchtabview.recentsearchesgridview.scrolledrecentsearchlist.checkdbssearchlink_0:rerunsearch/7F8452F12E5C433EPQ/None?site=sociologicalabstracts&t:ac=RecentSearches) |
| S5 | [MAINSUBJECT.EXACT("Special education") OR MAINSUBJECT.EXACT("Mainstreaming")](https://search.proquest.com/recentsearches.recentsearchtabview.recentsearchesgridview.scrolledrecentsearchlist.checkdbssearchlink:rerunsearch/A8DDBCA21A7F4447PQ/None?site=sociologicalabstracts&t:ac=RecentSearches) | [1,510](https://search.proquest.com/recentsearches.recentsearchtabview.recentsearchesgridview.scrolledrecentsearchlist.checkdbssearchlink_0:rerunsearch/A8DDBCA21A7F4447PQ/None?site=sociologicalabstracts&t:ac=RecentSearches) |
| S4 | [ti(integrat* OR immers* OR inclus* OR mainstream* OR placement*) OR ab(integrat* OR immers* OR inclus* OR mainstream* OR placement*)](https://search.proquest.com/recentsearches.recentsearchtabview.recentsearchesgridview.scrolledrecentsearchlist.checkdbssearchlink:rerunsearch/A8F75701369F4FEDPQ/None?site=sociologicalabstracts&t:ac=RecentSearches) | [98,474](https://search.proquest.com/recentsearches.recentsearchtabview.recentsearchesgridview.scrolledrecentsearchlist.checkdbssearchlink_0:rerunsearch/A8F75701369F4FEDPQ/None?site=sociologicalabstracts&t:ac=RecentSearches) |
| S3 | [S1 OR S2](https://search.proquest.com/recentsearches.recentsearchtabview.recentsearchesgridview.scrolledrecentsearchlist.checkdbssearchlink:rerunsearch/9710A6284D0E4ADDPQ/None?site=sociologicalabstracts&t:ac=RecentSearches) | [3,695](https://search.proquest.com/recentsearches.recentsearchtabview.recentsearchesgridview.scrolledrecentsearchlist.checkdbssearchlink_0:rerunsearch/9710A6284D0E4ADDPQ/None?site=sociologicalabstracts&t:ac=RecentSearches) |
| S2 | [MAINSUBJECT.EXACT("Learning disabilities") OR MAINSUBJECT.EXACT("Developmental disabilities")](https://search.proquest.com/recentsearches.recentsearchtabview.recentsearchesgridview.scrolledrecentsearchlist.checkdbssearchlink:rerunsearch/52D0A143C43745C4PQ/None?site=sociologicalabstracts&t:ac=RecentSearches) | [1,567](https://search.proquest.com/recentsearches.recentsearchtabview.recentsearchesgridview.scrolledrecentsearchlist.checkdbssearchlink_0:rerunsearch/52D0A143C43745C4PQ/None?site=sociologicalabstracts&t:ac=RecentSearches) |
| S1 | [ti(need* OR special* OR additional*) AND ti(student* OR pupil* OR child* OR youth* OR young*)](https://search.proquest.com/recentsearches.recentsearchtabview.recentsearchesgridview.scrolledrecentsearchlist.checkdbssearchlink:rerunsearch/D2FAFED607DE4BE7PQ/None?site=sociologicalabstracts&t:ac=RecentSearches) | [2,162](https://search.proquest.com/recentsearches.recentsearchtabview.recentsearchesgridview.scrolledrecentsearchlist.checkdbssearchlink_0:rerunsearch/D2FAFED607DE4BE7PQ/None?site=sociologicalabstracts&t:ac=RecentSearches) |

Social Science Citation Index & Science Citation Index. Search Performed 08/04/2021. Timespan=2000-2021.

| set | search | results |
| --- | --- | --- |
| S6 | #5 AND #4 AND #3 | 889 |
| S5 | TI=(effect* OR trial* OR experiment* OR control* OR random* OR impact* OR compar* OR difference*) OR AB=(effect* OR trial* OR experiment* OR control* OR random* OR impact* OR compar* OR difference*) | 19.178.775 |
| S4 | TI=(integrat* OR immers* OR inclus* OR mainstream* OR placement*) OR AB=(integrat* OR immers* OR inclus* OR mainstream* OR placement*) | [1.793.628](https://apps.webofknowledge.com/summary.do?product=WOS&doc=1&qid=14&SID=F4iWMx6jq3XvaV7OCe7&search_mode=AdvancedSearch&update_back2search_link_param=yes) |
| S3 | #2 AND #1 | [15.793](https://apps.webofknowledge.com/summary.do?product=WOS&doc=1&qid=21&SID=F4iWMx6jq3XvaV7OCe7&search_mode=CombineSearches&update_back2search_link_param=yes) |
| S2 | TI=(need* OR special* OR additional*) | [307.001](https://apps.webofknowledge.com/summary.do?product=WOS&doc=1&qid=20&SID=F4iWMx6jq3XvaV7OCe7&search_mode=AdvancedSearch&update_back2search_link_param=yes) |
| S1 | TI=(student* OR pupil* OR child* OR youth* OR young*) | [1.017.996](https://apps.webofknowledge.com/summary.do?product=WOS&doc=1&qid=17&SID=F4iWMx6jq3XvaV7OCe7&search_mode=AdvancedSearch&update_back2search_link_param=yes) |

## 3 Unavailable references

| **Adams Renard A. (2012). Data mining student special services information data to predict proficiency on high-stakes tests. . ProQuest Information & Learning.** |
| --- |
| **Arora Santosh, and Mishra Parul. (2008). A study on adjustment of children with visual impairment from special and integrated schools. GYAN, 4(2), pp.34-38.** |
| **Bakken Jeffrey P, Obiakor Festus E, and Rotatori Anthony F. (2012). Behavioral Disorders: Identification, Assessment, and Instruction of Students with EBD. Advances in Special Education. Volume 22. : Advances in Special Education (MS).** |
| **Bakken Jeffrey P, Obiakor Festus E, and Rotatori Anthony F. (2013). Learning Disabilities: Identification, Assessment, and Instruction of Students with LD. Advances in Special Education. Volume 24. : Advances in Special Education.** |
| **Bartolf Marcia M. (1977). Reading Achievement of Classified Students, Grades 7-8.. : , pp.. Available at: http://search.ebscohost.com/login.aspx?direct=true&db=eric&AN=ED181405&site=ehost-live.** |
| **Battista Alison Brennan. (2000). The impact of inclusion upon sixth graders' attitudes, self-esteem and academic performance. . ProQuest Information & Learning.** |
| **Brown Monica R. (2005). Adolescent Alienation: The Effects of Disability, Gender, and Ethnicity on Perceptions of School Life in Secondary Environments. Multiple Voices for Ethnically Diverse Exceptional Learners, 8(1), pp.17-35.** |
| **Deng Meng, and Manset Genevieve. (2000). Analysis of the "Learning in Regular Classrooms" Movement in China.. Mental Retardation, 38(2), pp.124-130.** |
| **Hintermair Manfred, Heyl Vera, and Janz Frauke. (2014). Exekutive funktionen und sozial-emotionale auffälligkeiten bei kindern mit verschiedenen formen von behinderung = Executive functioning, communicative competence, and behaviour disorders in students with different types of impairment. Vierteljahresschrift für Heilpädagogik und ihre Nachbargebiete, 83(3), pp.232-245.** |
| **Kaznowski Kimberly Lynn. (2003). A study comparing the school performance of slow learners who qualify for special education with slow learners who do not qualify for special education. . ProQuest Information & Learning.** |
| **Kocaj Aleksander, Kuhl Poldi, Kroth Anna J, Pant Hans Anand, and Stanat Petra. (2014). Wo lernen Kinder mit sonderpädagogischem Förderbedarf besser? Ein Vergleich schulischer Kompetenzen zwischen Regel- und Förderschulen in der Primarstufe.. Where do students with special educational needs learn better? A comparison of achievement between regular primary schools and special schools., 66(2), pp.165-191.** |
| **Kurth Jennifer. (2009). Academic outcomes in inclusive and non-inclusive special education programs for adolescents with autism spectrum disorders. . ProQuest Information & Learning.** |
| **McMillan Ian. (2004). Schools 'could do better' on inclusion.. Learning Disability Practice, 7(9), pp.5-5.** |
| **Rix Jonathan. (2008). Cracking the word code.. TES: Times Educational Supplement, (4772), pp.50-51.** |
| **Rohgalf Til. (2015). Die soziale Integration sprachauffälliger Kinder -- Ziele und Aufgaben inklusiver Pädagogik.. Social Integration of children with speech and language impairments in inclusive systems., 60(2), pp.66-76.** |
| **Rollins Lisa M. (2008). The influence of full inclusion on academics and self-concepts of students with learning disabilities. . ProQuest Information & Learning.** |
| **Schmidt Majda. (2000). Social integration of students with learning disabilities. Developmental Disabilities Bulletin, 28(2), pp.19-26.** |
| **Syngollitou Efi, and Louraki Eftyhia. (2005). Attendance in special classes and learning disabled children's self-esteem: A developmental approach. Psychology: The Journal of the Hellenic Psychological Society, 12(2), pp.210-231.** |
| **Tang Hua Cui. (2008). A Research and Analysis of the integrating disabled children in regular classes. . South China Normal University (People's Republic of China).** |
| **Waller Raymond J, and Conley Michael. (2005). Educational Placement of Children with Cochlear Implants. School Social Work Journal, 30(1), pp.75-83.** |
| **Zborteková Katarína. (2000). Effect of integrated education on cognitive and personality development of hearing-impaired children. Studia Psychologica, 42(3), pp.255-260.** |
| **Zborteková Katarína. (2000). Integrované vzdelávanie a kognitívny vývin sluchovo postihnutých detí = Integrated education and cognitive development of hearing-impaired children. Psychológia a Patopsychológia Dieťaťa, 35(1), pp.57-66.** |
| **Zeleiová Jaroslava Gajdošíková, and Bizová Naďa. (2016). QUALITY OF LIFE OF PUPILS WITH SENSORY IMPAIRMENT EDUCATED IN ORDINARY AND SPECIAL SCHOOLS.. International Multidisciplinary Scientific Conference on Social Sciences & Arts SGEM, , pp.1015-1022.** |

## 4 Additional searches

Additional searched were carried out in August and September 2021

| American Educational Research Association (AERA) | Advanced Search | "Special need students" | 9 |
| --- | --- | --- | --- |
|  |  | "special need students" AND placement | 9 |
|  |  | Special need students AND "placement" AND "Achievemen | 9 |
|  |  | "students with disabilities" AND placement | 0 |
|  |  | "students with disabilities" | 0 |
|  |  | "students with special educational needs" | 0 |
|  |  | special need AND student~ | 46 |
|  |  | disability~ AND student~ | 16 |
|  |  | "additional educational need" AND student~ | 2 |

| European Educational Research Association (EERA) | Search | "Special need students" | 1 |
| --- | --- | --- | --- |
|  |  | "Special need students" | 19 |
|  |  | "students with disabilities" AND "inclusion" | 48 |

| European Educational Research Association (EERA) American Educational Research Association (AERA)) | Advanced search (Abstract only) | "special needs students" AND placement | 6 |
| --- | --- | --- | --- |
|  | Advanced search (All fields) | "special needs students" AND placement AND inclusion AND mainstream AND effect (1996-2021) | 158 |
|  |  | "students with additional needs" AND placement | 19 |
|  |  |  |  |

| NBER working paper series | Search | "students with disabilities" AND "placement" | 29 |
| --- | --- | --- | --- |
|  |  | "special educational needs" AND inclusion | 14 |
|  |  | Mainstreaming OR "student placement" AND "student" AND "special" OR "additional" | 26 |
|  |  | students with disabilities | 77 |
|  |  | Special need AND placement | 48 |
|  |  | Student placement | 31 |
|  |  |  |  |
|  |  |  |  |

| OECD iLibrary | Advanced Search (All fields) | "students with disabilities" AND placement AND achievement AND effect | 192 |
| --- | --- | --- | --- |
|  |  | "students with additional needs" AND ‘placement’ | 14 |
|  |  | "special education student" AND placement AND achievement AND effect | 38 |
|  | Advanced Search (Abstract only) | "Special education student" | 0 |
|  |  | "students with disabilities" | 14 |
|  |  | "additional educational need" | 6 |
|  |  | "Special Needs Students" | 65 |
|  |  | Mainstreaming AND students | 19 |
|  |  | students with additional needs | 11 |
|  |  | Student AND Placement | 27 |
|  |  | Student AND "additional need" | 0 |

| Best Evidence Encyclopedia | Search | Special education student | 3 |
| --- | --- | --- | --- |
|  |  | Placement | 0 |
|  |  | Mainstreaming | 0 |
|  |  | additional educational need | 1 |
|  |  | students with disabilities | 0 |

| Academic publications from the university of Copenhagen |  | "Special education student" | 0 |
| --- | --- | --- | --- |
|  |  | Inclusive education | 0 |
|  |  | Education AND inclusion AND student | 23 |
|  |  | Special Needs Students | 6 |
|  |  | Student with disability | 8 |
|  |  | Mainstreaming education | 2 |
|  |  | Special education inclusive education | 7 |
|  |  | Inklusion AND elever | 4 |
|  |  | Elever med særlige behov | 1 |
|  |  | “Student Placement” | 10 |

| Social Care Online | Search | "Special education student" | 21 |
| --- | --- | --- | --- |
|  |  | "inclusive education" AND "special need" | 41 |
|  |  | inclusive education AND "student with disability" | 31 |
|  |  | "inclusive education" AND "special education" | 80 |
|  |  | students with learning disabilities | 47 |

| Cochrane Library | Advanced search (in Cochrane Reviews, Trials. Abstract) | "inclusive education" AND "special need" | 1 |
| --- | --- | --- | --- |
|  |  | "inclusive education" | 13 |
|  |  | "Special education student" | 7 |
|  |  | Education AND inclusion AND student | 18 |
|  |  | "students with disabilities" AND placement | 1 |
|  |  | "students with disabilities" | 18 |
|  |  | "students with special educational needs" | 3 |
|  |  | "Special Needs Students" | 3 |
|  |  | "additional educational need" | 1 |
|  |  | Mainstreaming AND education | 3 |
|  |  | Mainstreaming | 7 |
|  |  | "Special education" AND "inclusive education" | 2 |
|  |  | special AND education | 61 |

| EPPI‐Centre database of education research | Freetext search (Search history --> combine) | "special education" AND "students with disabilities" | 15 |
| --- | --- | --- | --- |
|  |  | Special Needs Students | 3 |
|  |  | "inclusive education" | 22 |
|  |  | "students with disabilities" | 59 |
|  |  | "students with special educational needs" | 1 |
|  |  | "Special need students" | 0 |
|  |  | additional educational need | 0 |
|  |  | "special education" AND "inclusive education" | 6 |
|  |  | Mainstreaming | 12 |
|  |  | Education AND inclusion AND student | 15 |
|  |  | general education classrooms | 34 |
|  |  | Emotional and behavioral difficulties | 3 |
|  |  | "behavioral difficulties" | 9 |
|  |  | "learning disabilities" AND "special education" | 22 |
|  |  | "learning disabilities" AND education | 54 |

| Campbell Journal of Systematic Reviews | Search | inclusive education | 5 |
| --- | --- | --- | --- |
|  |  | Special need student | 21 |
|  |  | learning disabilities | 12 |
|  |  | Mainstreaming | 0 |
|  |  | general education | 69 |
|  |  | special education | 69 |
|  |  | students with disabilities | 6 |
|  |  | learning disabilities | 12 |
|  |  | Student Placement | 2 |

| Google scholar | Advanced search | student special education inclusion. Med den nøjagtige sætning: special needs students AND special education | 13 |
| --- | --- | --- | --- |
|  |  | special education students AND inclusion | 11 |
|  |  | student inclusion "special education" | 100 |
|  |  | pupil inclusion "special education" | 100 |
|  |  | "special needs students" "mainstream education" "special education" | 100 |
|  |  | allintitle: student inclusion education placement OR OR OR inclusion OR OR OR "special need" "special education" | 17 |
|  |  | allintitle: student mainstream special education placement OR or OR mainstream OR or OR special OR need "special education" | 2 |

| Social science research network | advanced search | student inclusion "special education" | 8 |
| --- | --- | --- | --- |
|  |  | pupil inclusion "special education" | 0 |
|  |  | student mainstream "special education" | 0 |
|  |  | student "special education" | 65 |
|  |  | "students with disabilies" AND "special needs" AND inclusion | 1 |
|  |  | "students with disabilies" AND inclusion | 13 |
|  |  | mainstreaming "student placement" | 0 |
|  |  | mainstream "student placement" | 0 |
|  |  | "children with disabilities" mainstreaming | 0 |
|  |  | "children with disabilities" inclusion | 0 |
|  |  | "students with disabilities" mainstreaming | 0 |

| Skolporten- Swedish Dissertations | Search in : "videnskabelige tidsskrifter" | elev inkludering specialundervisning | 0 |
| --- | --- | --- | --- |
|  |  | elev inkludering | 3 |
|  |  | inkludering specialundervisning | 0 |
|  |  | inkludering funktionshinder | 0 |
|  |  | studerende inkludering | 0 |
|  |  | elev specialundervisning | 0 |
|  |  | specialundervisning | 1 |
|  |  | "special education" inclusion | 0 |
|  |  | "special education" | 3 |
|  |  | "students with disabilities" inclusion | 0 |
|  |  | mainstreaming students | 0 |
|  |  | student "special education" | 0 |

| DIVA - Swedish Digital Scientific Archives | advanced search, time interval: 2000-2021 | student AND inclusion AND "special education" | 35 |
| --- | --- | --- | --- |
|  |  | student AND mainstreaming AND "special education" | 0 |
|  |  | pupil AND inclusion AND "special education" | 16 |
|  |  | mainstreaming AND student placement | 0 |
|  |  | "student placement" AND "special education" | 0 |
|  |  | "students with additional needs" AND placement | 0 |
|  |  | "special needs students" AND "placement" | 1 |
|  |  | "special needs students" AND inclusion | 5 |
|  |  | inkludering AND speciaundervisning | 9 |
|  |  | inkludering AND elev AND specialundervisning | 5 |
|  |  | inkludering AND elev AND funktionshinder | 3 |

| NORA - Norweigan Open Research Archives |  | student AND inclusion AND "special education" | 13 |
| --- | --- | --- | --- |
|  |  | student AND mainstreaming AND "special education" | 4 |
|  |  | pupil AND inclusion AND "special education" | 4 |
|  |  | "student placement" AND "special education" | 0 |
|  |  | "students with additional needs" AND placement | 0 |
|  |  | "special needs students" AND "placement" | 0 |
|  |  | "special needs students" AND inclusion | 0 |
|  |  | inkludering AND elev AND specialundervisning | 23 |

| SwePub—Academic publications at Swedish universities | Search in titles | student AND inclusion AND "special education" | 1 |
| --- | --- | --- | --- |
|  |  | student AND mainstreaming AND "special education" | 0 |
|  |  | pupil AND inclusion AND "special education" | 11 |
|  |  | "student placement" AND "special education" | 0 |
|  |  | "students with additional needs" AND placement | 0 |
|  |  | "special needs students" AND "placement" | 2 |
|  |  | "special needs students" AND inclusion | 4 |
|  |  | inkludering AND elev AND "specialundervisning" | 2 |
|  |  | inkludering AND elev AND "speciella behov" | 0 |
|  |  | inkludering AND speciaundervisning | 4 |
|  | free text search | student AND inclusion AND "special education" | 21 |

| AAU Publications—Academic publications from the University of Aarhus |  | student AND inclusion AND "special education" | 4 |
| --- | --- | --- | --- |
|  |  | student AND mainstreaming AND "special education" | 7 |
|  |  | pupil AND inclusion AND "special education" | 1 |
|  |  | "student placement" AND "special education" | 0 |
|  |  | "students with additional needs" AND placement | 0 |
|  |  | "special needs students" AND "placement" | 0 |
|  |  | "special needs students" AND inclusion | 0 |
|  |  | inkludering AND elev AND "specialundervisning" | 1 |
|  |  | inklusion AND elev AND specialundervisning | 10 |
|  |  | diagnose inklusion børn | 16 |
|  |  | inkludering AND elev AND "speciella behov" | 0 |
|  |  | inkludering elev funktionshinder | 0 |

| Centre for Reviews and Dissemination |  | student AND inclusion AND "special education" | 7 |
| --- | --- | --- | --- |
|  |  | student AND mainstreaming AND "special education" | 0 |
|  |  | pupil AND inclusion AND "special education" | 0 |
|  |  | "student placement" AND "special education" | 0 |
|  |  | "students with additional needs" AND placement | 0 |
|  |  | "special education" AND inclusion | 45 |
|  |  | "special needs students" AND inclusion | 0 |
|  |  | disability AND student AND inclusion | 15 |

| Google Searches | Advanced search, title | inclusion AND "special education" AND student | 155 |
| --- | --- | --- | --- |
|  | Advanced search, title | student AND mainstreaming AND "special education" | 7 |
|  | Advanced search, title | inclusion AND pupil AND "special education | 1 |
|  | Advanced search, title | "student placement" AND "special education" | 1 |
|  | Advanced search, title | "students with additional needs" AND "special education" | 1 |
|  | Advanced search, title | "students with additional needs" AND "placement" | 0 |
|  | Advanced search, title | "special needs students" AND "placement" | 4 |
|  | Advanced search, title | inclusion AND "special needs students AND special education" | 114 |

| Open Grey | Advanced searches | "Students with disabilities" AND inclus* | 2 |
| --- | --- | --- | --- |
|  |  | "Students with disabilities" AND placement* | 0 |
|  |  | "Students with disabilities" AND integrat* | 2 |
|  |  | "students with disabilities" AND mainstream* | 1 |
|  |  | students with disabilities AND "academic accomodations" | 0 |
|  |  | "students with disabilities" AND immers* | 0 |
|  |  | "Special needs students" AND inclus* | 0 |
|  |  | "Special needs students" AND placement* | 0 |
|  |  | "Special needs students" AND integrat* | 0 |
|  |  | "Special needs students" AND mainstream* | 0 |
|  |  | "Special needs students" AND "academic accomodations" | 0 |
|  |  | "Special needs students" AND immers* | 0 |
|  |  | Special* AND student* AND inclus* | 36 |
|  |  | Special* AND student* AND mainstream* | 21 |
|  |  | Special* AND student* AND integrat* | 42 |
|  |  | Special* AND student* AND placement* | 9 |
|  |  | Special* AND student* AND immers* | 2 |
|  |  | Special* AND student* AND "academic accomodations" | 0 |
|  |  | Special* AND child* AND "academic accomodations" | 0 |
|  |  | Special* AND child* AND immers* | 3 |
|  |  | Special* AND child* AND placement* | 14 |
|  |  | Special* AND child* AND integrat* | 53 |
|  |  | Special* AND child* AND mainstream* | 59 |
|  |  | Special* AND child* AND inclus* | 60 |
|  |  | Special* AND pupil* AND inclus* | 31 |
|  |  | Special* AND pupil* AND mainstream* | 42 |
|  |  | Special* AND pupil* AND integrat* | 15 |
|  |  | Special* AND pupil* AND placement* | 6 |
|  |  | Special* AND pupil* AND immers* | 2 |
|  |  | Special* AND pupil* AND "academic accomodations" | 0 |
|  |  | additional* AND pupil* AND "academic accomodations" | 0 |
|  |  | additional* AND pupil* AND immers* | 3 |
|  |  | additional* AND pupil* AND placement* | 1 |
|  |  | additional* AND pupil* AND integrat* | 7 |
|  |  | additional* AND pupil* AND mainstream* | 5 |
|  |  | additional* AND pupil* AND inclus* | 5 |
|  |  | special* AND young* AND inclus* | 16 |
|  |  | special* AND youth* AND inclus* | 3 |
|  |  | special* AND youth* AND mainstream* | 1 |
|  |  | special* AND youth* AND integrat* | 9 |
|  |  | special* AND youth* AND placement* | 3 |
|  |  | special* AND youth* AND immers* | 0 |
|  |  | special* AND youth* AND "academic accomodations" | 0 |
|  |  | special* AND young* AND "academic accomodations" | 0 |
|  |  | special* AND young* AND immers* | 1 |
|  |  | special* AND young* AND placement* | 7 |
|  |  | special* AND young* AND integrat* | 28 |
|  |  | special* AND young* AND mainstream* | 19 |

| [Basic Search - ProQuest](https://www.proquest.com/) | advanced search | ti(inclus* OR integrat* OR placement* OR immers* OR mainstream*) AND (student* OR pupil* OR child* OR youth* OR young* OR educational*) Additional limits: Date from 2000-2022, Source type: Dissertations & Theses | 1718 |
| --- | --- | --- | --- |
